# Molecular Interactions within Nanoconfinement of Model DNA Nanostructures Controlled by Compensatory Kinetics as Revealed by Single-Molecule Fluorescence Analysis

**DOI:** 10.1021/jacsau.5c00774

**Published:** 2025-08-28

**Authors:** Nora Hagleitner-Ertuğrul, Yongzheng Xing, Juergen Pfeffermann, Alexia Rottensteiner, Anna Gaugutz, Denis G. Knyazev, Peter Pohl, Stefan Howorka

**Affiliations:** † Institute of Molecular Biophysics and Membrane Biophysics, 27266 Johannes Kepler University Linz, Gruberstraße 40, 4020 Linz, Austria; ‡ National Engineering Research Center for Colloidal Materials, School of Chemistry and Chemical Engineering, 12589 Shandong University, 27 Shanda South Road, Jinan 250100, China; § Department of Chemistry, Institute of Structural Molecular Biology, 4919 University College London, London WC1H 0AJ, United Kingdom; ∥ Institute of Applied Physics, TU Wien, Wiedner Hauptstraße 8-10, 1040 Vienna, Austria

**Keywords:** molecular recognition, confinement, DNA nanopore, dc-FCCS

## Abstract

Molecular interactions under steric confinement are important in chemistry, biology, biotechnology, and medicine. The impact of nanoconfinement on the underpinning kinetics and affinities is, however, unclear. While theoretical frameworks predict any effect only for very fast diffusion-limited association kinetics, experimental studies report that molecules can be trapped inside confined spaces to increase the effective local concentration and impact binding kinetics. Understanding is furthermore complicated by poorly comparable confinement geometries and reactions. Here, we determine the kinetics and affinities for interactions slower than the diffusion limit using highly modular DNA origami nanopores as model nanoconfinement systems. The pores feature either inside or outside their narrow lumen a single receptor, which can bind to three differently sized biomolecular ligands. We conduct kinetic binding analysis at the single-molecule resolution using fluorescence correlation spectroscopy to readily acquire large datasets and help overcome limitations of other single-molecule approaches. Nanoconfinement is found to hinder ligand association and dissociation, even below the diffusion limit. Yet, both suppressed kinetics compensate for each other to yield the same overall equillibrium affinity as nonconfined receptors, while local concentration enhancement by ligand trapping was not observed. We expect our insights and experimental strategy to guide the development of biosensing nanopores and help advance the understanding of biological nanochannels.

## Graphical abstract

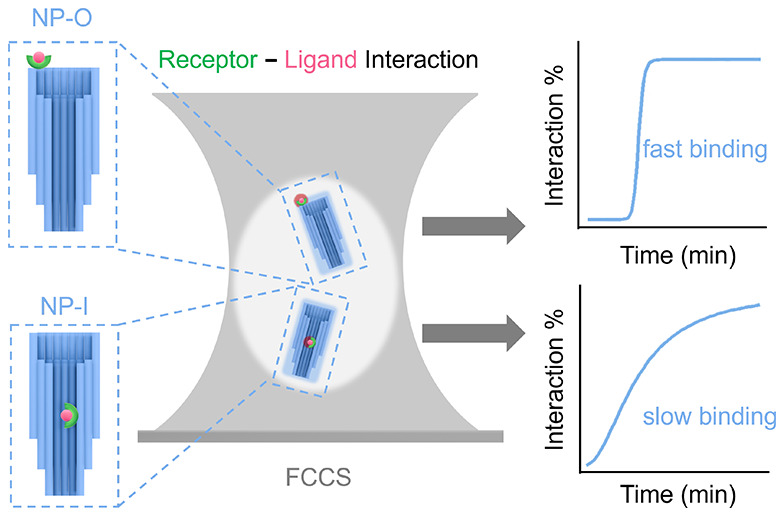

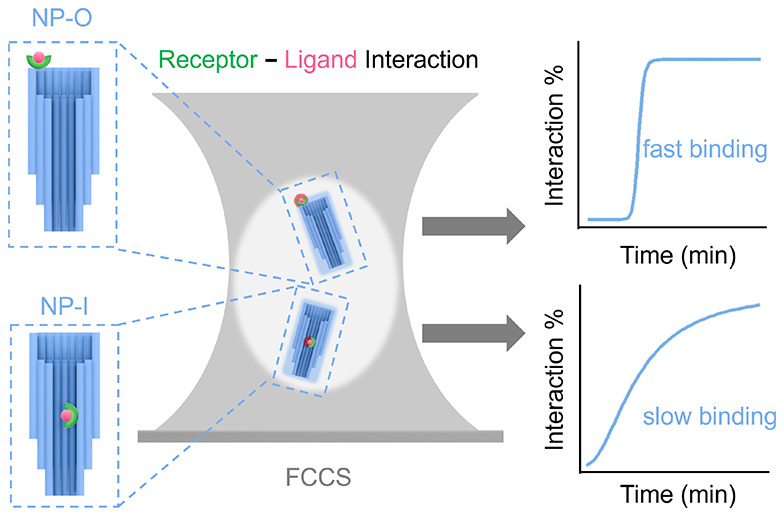

## Introduction

Molecular interactions under steric confinement are key in chemistry,^1^ biology,^2,3^ biotechnology,^4,5^ and biomedicine. In its simplest form, nanoconfinement limits ligand access to recessed receptor sites but can lead to localized rebinding and directional ligand movement in more complex systems with multiple receptor sites, often positioned within the hollow lumen of bilayer-spanning biological channels or nanopores.^6–11^ Examples of nanoconfinement in biology include eukaryotic ligand-gated ion channels,^12^ G-protein coupled receptors,^13^ and drug-resistance conferring ABC transporters,^14,15^ but also bacterial maltoporin and related porins,^16^ and the large nuclear pore complex,^8^ the lumen of which is filled with a meshwork of receptors to directionally transport cargo across the nuclear membrane. Within biotechnology, nanopores with engineered receptor sites in the pore lumen sense metal ions,^17,18^ small molecules,^19^ proteins,^20^ enantiomers,^21,22^ and nucleotides,^23^ and are widely used for portable DNA/RNA^4,24^ and peptide^25,26^ sequencing. Additionally, porous membranes with intrapore molecular binding sites are used for affinity-based purification.^27,28^ Given the wide range of molecular interactions, understanding the influence of confinement on these interactions is of fundamental scientific interest and provides a rational design basis for improved nanopores with better selectivity and transport performance. To maximize insight, interaction kinetics and binding affinities under nanoconfinement should be known in comparison to nonconfining but otherwise identical conditions.

Despite its importance, the impact of nanoconfinement on kinetics is currently unsettled. Theoretical considerations predict that confinement should only affect interactions with diffusion-limited association kinetics.^29–31^ By contrast, experimental reports describe the up-concentration of ligands and accelerated kinetics for receptors within confinement, even at rates below the diffusion limit.^32–37^ These findings may be caused by conformational restriction on movement within nanoconfinement or the rebinding to multiple receptors.^38–40^ Furthermore, comparing kinetic findings from different reports is difficult given the fragmented overlap between varying experimental systems with differing confinement dimensions, receptor types and positions, and ligand sizes.^32–35,41^ Several of the fundamental kinetic questions could be answered using a defined modular system featuring a single receptor inside and outside a sterically controlled nanoconfinement, something which is challenging to engineer with typically used inorganic^42,43^ or protein-based^44,45^ materials. The suitable modular systems could, however, be made with DNA nanostructures of programmable nanoscale dimensions and shape, as well as tunable receptor position and types,^46–52^ but DNA nanotechnology has not yet been exploited for kinetic analysis to answer the above questions.

Clear insight into nanoconfined kinetics may also be achieved with a suitable single-molecule technique, given that ensemble methods often mask important aspects of molecular interaction. In classical and powerful single-molecule force microscopy (SMFM), a molecular ligand is placed at the tip of a cantilever and oscillated to enable repeated interaction with a molecular receptor adsorbed on a solid substrate. SMFM measures binding kinetics and rupturing forces of molecular interactions^53^ under nonconfining conditions^54–56^ and in nanoconfinement by using ligands tethered via a long flexible linker to the tip in order to reach into the channel lumen.^41,57,58^ Analysis via SMFM is, however, limited by issues stemming from the channels’ immobilization on nonbiological solid substrates^59^ such as poor adhesion, channel deformation or misaligned orientation to the cantilever oscillation, and poor molecular accessibility of intrachannel receptor sites leading to insufficient, biased, or challenging-to-interpret binding kinetics.^41,60–62^ In single-channel current recordings, by comparison, biological channels are in their native bilayer environment to track the reversible binding of ligands inside the lumen in real time by measuring fluctuations in ion current flowing through the channel.^20,63,64^ However, ligand binding outside the channel lumen is usually not directly detectable, and current recordings can suffer from low throughput, while the binding kinetics can be biased by the strong ionic flux across the channel lumen.^65–67^ Another single-molecule technique, dual-color fluorescence cross-correlation spectroscopy (dc-FCCS), measures the correlated motion of two distinct fluorescently labeled and usually water-soluble molecular species at high throughput and can, in principle, overcome limitations faced by SMFM and current recordings. Indeed, dc-FCCS has helped elucidate binding affinity and binding kinetics for molecular interactions in vitro^68–73^ and in vivo,^74–77^ but it is rarely applied for studying molecular interactions under nanoconfinement within biological channels.^78,79^

In this study, we elucidate confinement effects on the binding kinetics of ligand–receptor interactions using dc-FCCS readout in combination with a model nanopore composed of DNA. DNA nanopores^80,81^ made from bundled DNA duplexes provide an ideal platform^46,47,82–86^ by offering precise control over channel dimensions,^41,87,88^ receptor type,^41,89^ and receptor positioning,^41^ which are parameters ideally tunable in an optimal model system for nanoconfinement yet difficult to flexibly control in biological protein channels. Additionally, fluorescent labels can be easily introduced with precise control over type, number, and position, which is a prerequisite for analysis with dc-FCCS. Our custom-made nanopore^90^ has a 7.5 × 7.5 nm-wide and 46 nm-high pore lumen (Figure 1A), where receptors are engineered inside and outside the lumen (Figure 1B) to enable binding of a variety of ligands including DNA and proteins (Figure 1C) to systematically cover the influence of ligand size. All ligand–receptor interactions are chosen to have binding kinetics below the diffusion limit. We use the water-soluble DNA nanopore for dc-FCCS measurements in solution (Figure 1D), which eliminates the need for their immobilization on nonbiological surfaces or reconstitution into lipid vesicles.

**Figure 1:**
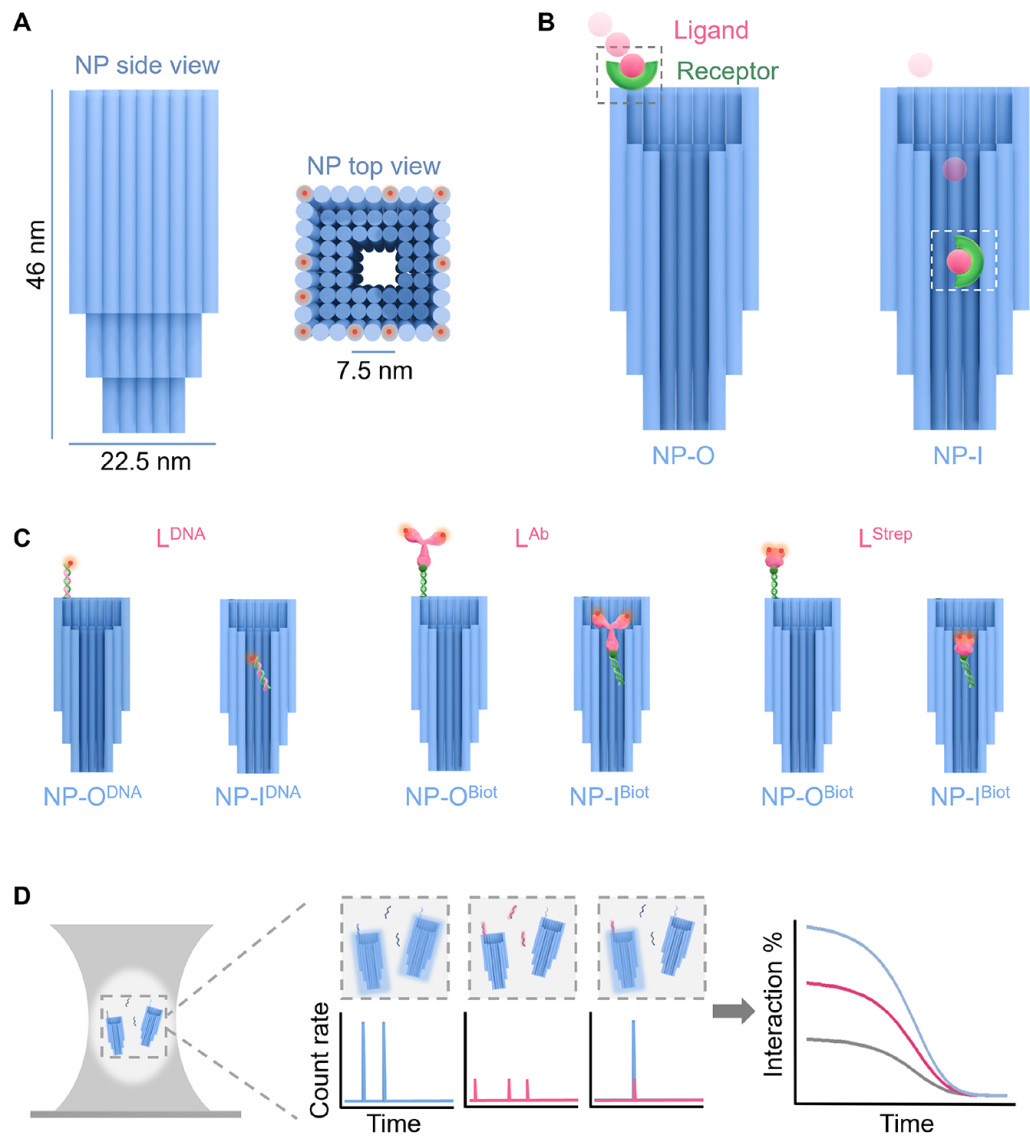
Schematic drawing of the DNA nanopores used to study molecular interaction between receptors and ligands under nanoconfinement using dual-color fluorescence cross-correlation spectroscopy. (A) Overall dimensions of the nanopore (NP) composed of bundled DNA duplexes (blue cylinders) in side view (left) and top view (right), which also shows the positions of the Alexa Fluor 647 fluorophores as orange halos. (B) Interaction of a ligand with a receptor located either at nanopore type NP-O outside nanoconfinement or at NP-I within the confining pore lumen. Ligand (magenta) and receptor (green) are drawn schematically. (C) Nanopores NP-O^DNA ^and NP-I^DNA^ carrying a single-stranded DNA strand (left, green) which binds ligand L^DNA^ (magenta) composed of a short ssDNA. Nanopores NP-O^Biot^ and NP-I^Biot^ with a biotin tag (dark green dot at a green duplex tether) capture medium-sized streptavidin (L^Strep^, right) or a large antibody (L^Ab^, middle). The Alexa Fluor 488 fluorophores on the ligands are indicated by orange halos. For ligands L^Ab^ and L^Strep^, the position and number of fluorophores are approximate due to the random modification of these protein ligands. (D) Detecting nanopore–ligand interactions as they diffuse through the hourglass-shaped confocal volume of dc-FCCS (left). As shown in the middle panels, fluctuations in the fluorescent signal of NP (free and binary complex NP·L) and ligand (free and NP·L) are detected in their respective fluorescence windows, while complex NP·L gives rise to the co-occurrence of fluorescent signals in both detection windows. As illustrated on the right, fluorescent intensity traces are converted to autocorrelation curves (blue for NP, magenta for L) and cross-correlated for NP·L (gray).

The research highlights of our study are, first, that nanoconfinement surprisingly and strongly affects ligand association and dissociation kinetics to internal single receptors, despite the slower-than-diffusion rates. The kinetics do not suggest any ligand up-concentration found for other systems. Second, our kinetic slow-down effects strikingly compensate for each other to yield the same overall affinity compared to nonconfined receptors. Third, dc-FCCS and DNA nanostructures are established as powerful and versatile tools in providing easy-to-access, high-data-number, and reliable kinetic analysis of molecular interactions in and out of nanoconfinement. Finally, our dc-FCCS-based approach for the kinetic analysis of confinement may be extended to biological channels, which can conveniently be solubilized with membrane nanodiscs.^91^

## Results and Discussion

### Design of the DNA Nanopore

The DNA nanopore, termed NP, is composed of 72 parallel, aligned DNA duplexes (Figure 1A, blue cylinders), which are oriented in a square lattice fashion and interlinked by DNA crossovers (Figure S1). The design of the DNA nanopore harnesses the principle of DNA origami, where the DNA nanostructure is composed of a long single-stranded scaffold strand and shorter staple strands to form interlinked hybridized sections. Using the caDNAno software,^92^ we designed NP to be 46 nm in height and 22.5 nm in outer width. The inner lumen is 7.5 × 7.5 nm in the narrow section but wider at the top entrance (Figure 1A). Corresponding to the varying lumen width, the pore wall ranges in thickness from 3 duplexes to 1 duplex (Figures 1A and 1B).

Within the structural framework of the NP, a single molecular receptor was placed inside the narrow lumen to yield pore type NP-I (Figure 1B, right). This pore helped probe the influence of nanoconfinement by carrying the receptor at a distance of 25 nm from the pore entrance. As a control for lack of confinement, the single receptor was alternatively positioned outside the lumen at the pore entrance to obtain NP-O (Figure 1B, left). Two different types of molecular receptors were attached. The first was composed of a single-stranded DNA of 20 nucleotides (nt) in length (Figure 1C, left). The resulting pore variants carrying DNA in the inner and outer positions were termed NP-I^DNA^ and NP-O^DNA^, respectively (Figure 1C, left and Table S1). The second receptor was biotin at the terminus of a DNA oligonucleotide hybridized to the DNA receptor; the corresponding pores are referred to as NP-I^Biot^ and NP-O^Biot^ (Figure 1C, middle, right and Table S1).

To maximize the insight into nanoconfinement effects, we used ligands of varying sizes. The first ligand (L) was the small, 20 nt-long ssDNA, termed L^DNA^, with a length of 6.3 nm when elongated (Tables S2 and S3) and capable of binding to NP-I^DNA^ or NP-O^DNA^ (Figure 1C, left). The other two ligands were the medium-sized streptavidin L^Strep^ (5.5 × 5.5 × 5.5 nm)^93^ and the larger antibiotin antibody L^Ab^ (14.5 × 8.5 × 4.0 nm)^94^ for binding to biotinylated pores NP-I^Biot^ and NP-O^Biot^ (Figure 1C, right, middle). Both ligands and the nanopores were carrying fluorophore tags to enable detection with dc-FCCS. The fluorophore for the ligands was Alexa Fluor 488 (AF488) placed distant to the ligands’ binding parts, while NP was decorated with 10 Alexa Fluor 647 (AF647) tags outside of the pore lumen (Figure 1A and Figure S2). Details on assembly of the DNA nanopores and their characterization with gel electrophoresis and transmission electron microscopy, confirmation of their fluorescence labeling with gel electrophoresis and FCS, comparative analysis of experimentally found nanopore dimensions with canDO and oxDNA simulations, and FCS analysis of a dye-labeled ligand are provided in the Supporting Information comprising Tables S4–S7 and Figures S3–S10.

### Tracking Ligand Binding to Receptor Pores with Dual-Color Fluorescence Cross-Correlation Spectroscopy

We used dc-FCCS to quantify interactions between fluorescently labeled ligands and receptors in DNA nanopores. Fluorescence signals from the ligand and nanopore were detected within overlapping femtoliter-sized confocal volumes excited by two lasers (488 and 633 nm, Figures 1D and S11). Three species were recorded: free NP, free L, and complex NP·L, with binding indicated by coincident fluorescence spikes in both detection channels (Figure 1D). Fluorescence intensity traces were analyzed by the autocorrelation function *G*(τ), which measures the temporal self-similarity of fluorescence signals and reveal diffusion times.^95–97^ Cross-correlation quantifies codiffusion of NP and L, indicating complex formation.^69,98^ We determined the percentage of NP bound, denoted as CCP, by the following equation and values of the respective fitted correlation function at τ = 0 (refs 69 and 99)­

$$\mathrm{CCP}=\frac{G_{\mathrm{NP}\cdot L}}{G_{L}}\cdot 100$$ 1

Further details on FCS and dc-FCCS are provided in the Supporting Information, Section 1.7.

### Reversible DNA Binding to the Nanoconfined Receptor Inside the Nanopore Controlled by Compensatory Kinetics

We explored any influence of nanoconfinement by determining the kinetics for binding of the DNA ligand L^DNA^ to its DNA receptor within nanopore NP-I^DNA^ (Figure 2A, bottom). Considering steric dimensions, we expected that L^DNA^ with an extended length of 6.3 nm and hydrodynamic diameter of 2.0 nm would be able to diffuse into the nanopore lumen of 7.5 × 7.5 nm and reversibly bind to the complementary single-stranded DNA receptor. As a control, we used the nonconfining NP-O^DNA^, which carries the DNA receptor at the pore entrance (Figure 2A, top). Considering its length and base sequence, DNA ligand L^DNA^ was anticipated to bind to the DNA receptor strand with an equilibrium dissociation constant *K*_d_ of approximately 10^–9^ M (refs 71 and 100).

**Figure 2:**
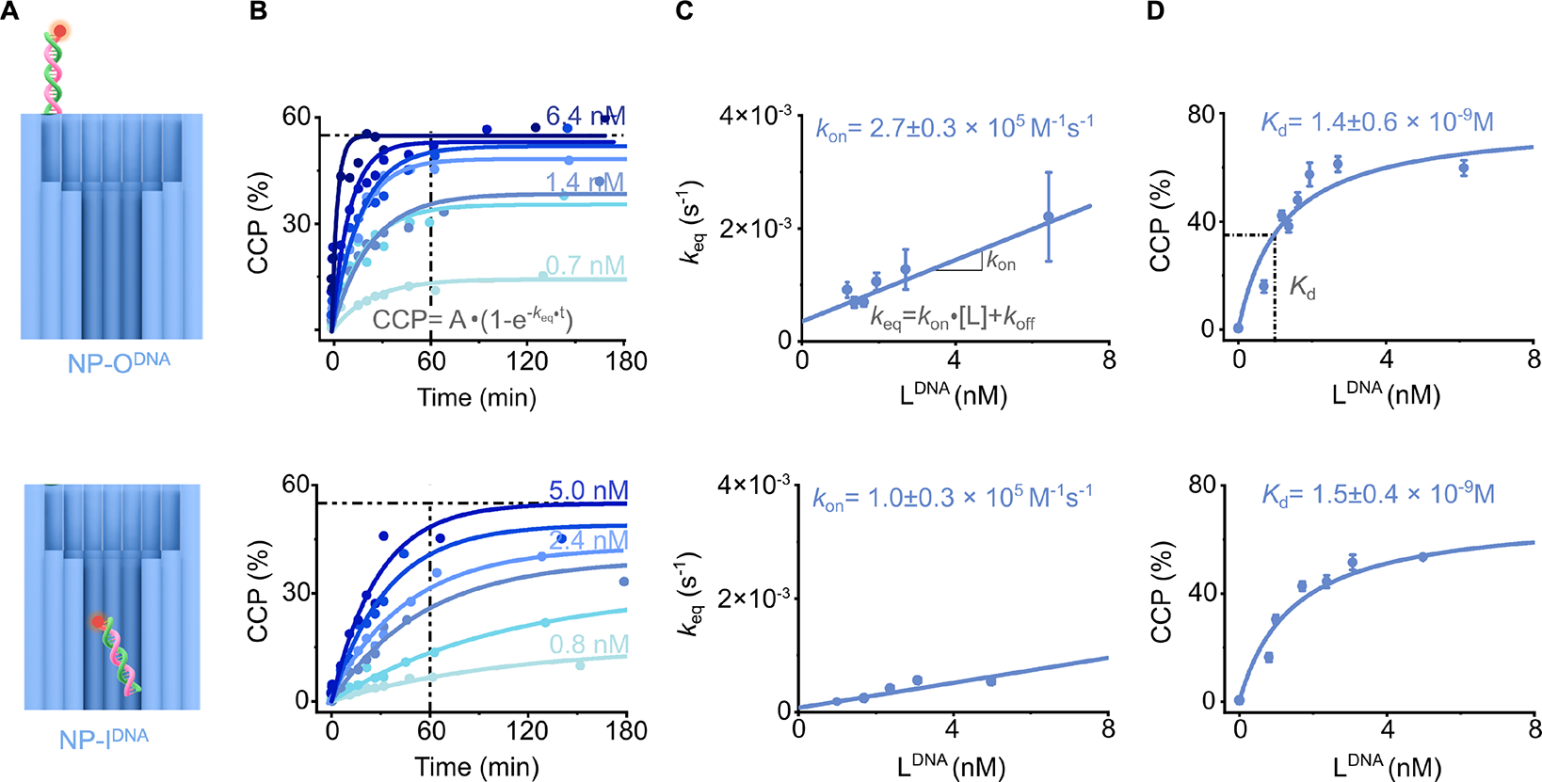
Binding affinities and binding kinetics of DNA–DNA hybridization within and out of nanoconfinement. (A) Schematic drawing of the interaction of L^DNA^ with NP-O^DNA^ (top) and NP-I^DNA^ (bottom). (B–D) Data for NP-O^DNA^ (top) and NP-I^DNA^ (bottom). (B) Kinetic traces for the binding of the NP variants to L^DNA^, plotted as CCP as a function of incubation time, and at different L^DNA^ concentrations. CCP measures the percentage of the binary NP·L complex relative to the total NP. (C) Plots of equilibrium rate constant *k*_eq_—obtained from the exponential fits to graphs in (B)—versus L^DNA^ concentration to obtain *k*_on_ from the slope of the linear fit. (D) Plots of CCP—after 180 min equilibration of binding—versus L^DNA^ concentration to obtain *K*_d_ via the Langmuir–Hill fit. For NP-O^DNA^, L^DNA^ concentrations were 0.7, 1.2, 1.4, 1.6, 1.9, 2.7, and 6.4 nM, and for NP-I^DNA^, the concentrations were 0.8, 1.0, 1.7, 2.4, 3.1, and 5.0 nM. The concentration of nanopores was 1.3 nM.

We first kinetically tracked the binding of ligand L^DNA^ to nanopore NP-O^DNA^ (Figure 2A, top) to set the reference point for the absence of nanoconfinement. Using dc-FCCS, we measured CCP, which quantifies the fraction of the binary complex NP-O^DNA^·L^DNA^, for a time window of up to 180 min (Figure 2B, top). The kinetic traces were also obtained for a range of ligand concentrations to gain broader insights into the interaction. As seen from the kinetic traces (Figure 2B, top), reversible ligand binding was dependent on both incubation time and ligand concentration, whereby longer duration and higher concentration increased the binding extent of CCP (Figure 2B, top). For example, binding at the lowest ligand concentration of 0.7 nM reached a plateau after 60 min, while it took only 20 min at the highest concentration of 6.4 nM (Figure 2B, top). The maximum degree of binding at a CCP value of around 60% is set by the optical overlap between the confocal volumes detecting the nanopore and ligand, which was determined prior to experiments using a fluorescently double-labeled DNA duplex (Table S8).^71^

To probe for any influence of nanoconfinement, we kinetically tracked the binding of ligand L^DNA^ to nanopore NP-I^DNA^ under otherwise identical conditions, such as ligand concentration range and incubation time. Comparison of the resulting traces for NP-I^DNA^ (Figure 2B, bottom) with those for NP-O^DNA^ (Figure 2B, top) revealed striking differences. Binding to the nanoconfined receptor in NP-I^DNA^ was considerably slower than that for the more accessible DNA in NP-O^DNA^ as it took up to 120 min to reach equilibrium (Figures 2B. For further analysis, the kinetic profiles were fitted with^101^

$$\mathrm{CCP}=A\cdot (1-\exp^{-k_{\mathrm{eq}}\cdot t})$$ 2

where the pre-exponential factor *A* represents the maximum binding extent, *t* is the incubation time, and *k*_eq_ is the rate constant for reaching the equilibrium of reversible binding.

To obtain more quantitative kinetic information, we determined the kinetic association rate constant *k*_on_ for reversible DNA duplex formation to the DNA receptor in NP-O^DNA^ as well as NP-I^DNA^. For both cases, the rate constant *k*_on_ was derived from a linear fit to a plot of the *k*_eq_ values against the corresponding ligand concentrations [L], using^101^

$$k_{\mathrm{eq}}=k_{\mathrm{on}}\cdot [L]+k_{\mathrm{off}}$$ 3

where *k*_off_ is the dissociation rate constant. The slope of the plot for eq 3 (Figure 2C, top) was used to obtain the association rate constant *k*_on_. The resulting *k*_on_ value for NP-I^DNA^ was 1.0 ± 0.3 × 10^5^ M^–1^ s^–1^, which is lower than for NP-O^DNA^ at 2.7 ± 0.3 × 10^5^ M^–1^ s^–1^ (Table 1).

**Table 1:** Binding Affinities and Binding Kinetics of Receptor–Ligand Interactions^a^

ligand	NP	*K*_d_ (M)	*k*_on_ (M^–1^ s^–1^)	*k*_off_ (s^–1^)
L^DNA^	NP-O^DNA^	1.4 ± 0.6 × 10^–9^	2.7 ± 0.3 × 10^5^	3.9 ± 1.7 × 10^–4^
	NP-I^DNA^	1.5 ± 0.4 × 10^–9^	1.0 ± 0.3 × 10^5^	1.5 ± 0.6 × 10^–4^
L^Ab^	NP-O^Biot^	3.4 ± 0.9 × 10^–8^	3.3 ± 1.1 × 10^5^	1.1 ± 0.2 × 10^–2^
	NP-I^Biot^	3.2 ± 1.1 × 10^–8^	1.7 ± 0.6 × 10^5^	0.54 ± 0.06 × 10^–2^
L^Strep^	NP-O^Biot^	2 ± 30 × 10^–12^	1.2 ± 0.1 × 10^6^	-
	NP-I^Biot^	4 ± 25 × 10^–12^	1.0 ± 0.2 × 10^6^	-

a For L^DNA^, *k*_off_ was calculated from experimentally determined *K*_d_ and *k*_on_. For L^Ab^, *k*_on_ was calculated from experimentally determined *K*_d_ and *k*_off_.

The 2.7-fold lower rate constant for binding within the confined lumen of NP-I^DNA^ (Figure 2C, bottom) is surprising as theoretical biophysical considerations^29–31,102–106^ hold that nanoconfinement should only influence molecular interactions that are diffusion-controlled, with *k*_on_ values at 10^7^ – 10^8^ M^–1^ s^–1^. While this is not the case for DNA hybridization, our observed nanoconfinement effect is real and demonstrates the analytical power of dc-FCCS to detect subtle differences in a kinetic regime close to diffusion control. In molecular terms, the 2.7-fold slower association rate constant likely reflects the steric hindrance by nanoconfinement, which can obstruct the binding of the DNA ligand to the receptor site buried inside the lumen of NP-I^DNA^. Our kinetic rate constants are in line with values for comparable DNA hybridization interactions determined with other techniques.^107–110^ We note that eq 3 suggests that *k*_off_ may also be obtained from the extrapolated intercept with the *y*-axis (Figure 2C). However, according to the literature, this is associated with too large an error.^101^

To confirm that the dc-FCCS-measured codiffusion of the ligand and the receptor-nanopore only occurs upon the successful binding of both, we carried out two negative control experiments. In the first, a ligand and nanopore without a receptor did not show cross-correlation signals (Figure S13) wich is in line with expectations. Similarly, in the second control, a DNA ligand mismatched in sequence to the receptor (Table S3) within the pore lumen did not cause cross-correlation (Figure S14), consistent with NUPACK analysis confirming the absence of hybridization (Figure S15). In addition, we evaluated the structural stability of the nanopore lumen with oxDNA simulations (Figures S6 and S7) and established that the pore diameter remains constant over time, thereby excluding the possibility that the ligand is transiently trapped due to structural fluctuations.

To further analyze DNA hybridization in and out of nanoconfinement, we determined the equilibrium dissociation constant *K*_d_ for NP-I^DNA^ and NP-O^DNA^. Therefore, we plotted the degree of binding of CCP, as obtained after 3 h of incubation, as a function of L^DNA^ concentration (Figure 2D). The plots were then fitted to the Langmuir–Hill isotherm^101^ with

$$\mathrm{CCP}=A\cdot \frac{\left[L\right]}{[L]+K_{d}}$$ 4

where *A* is the maximum degree of binding. The extracted value of *K*_d_ is 1.5 ± 0.4 × 10^–9^ M for NP-I^DNA^ and 1.4 ± 0.6 × 10^–9^ M for NP-O^DNA^ (Table 1). The similarity of both values highlights that the equilibrium affinities for binding in and out of the nanoconfinement are quite similar. The similarity of *K*_d_ values contrasts with the observed slower *k*_on_ kinetics for binding inside confinement than binding out of confinement (Table 1). This discrepancy was resolved by calculating the dissociation rate constant *k*_off_ from *K*_d_ and *k*_on_ using

$$K_{d}=\frac{k_{\mathrm{off}}}{k_{\mathrm{on}}}$$ 5

to yield *k*_off_ values of 1.5 ± 0.6 × 10^–4^ s^–1^ for NP-I^DNA^ and 3.9 ± 1.7 × 10^–4^ s^–1^ for NP-O^DNA^ (Table 1). The 2.6-fold slower dissociation kinetics likely reflects the steric hindrance of nanoconfinement in obstructing the dissociation of the DNA ligand from the receptor site buried inside the lumen of NP-I^DNA^.

Taken together, the kinetic and equilibrium data highlight that steric hindrance in nanoconfinement leads to lower association kinetics as well as to lower dissociation kinetics, but that the two kinetic trends compensate for each other to yield an equilibrium *K*_d_, which is within error of the binding constant outside nanoconfinement. It is unlikely that the kinetics are strongly influenced by electrostatic repulsion between the negatively charged DNA ligand and the negatively charged DNA nanolumen, given the use of an electrostatically screening buffer containing 10 mM MgCl_2_.

### Antibody Binding to Biotin Confirms the Nanoconfinement Effect and Yields Kinetics for Defined Monovalent Binding

After establishing confinement effects on association and dissociation for a small DNA ligand, we next probed whether a considerably larger antibody leads to similar or even size-proportional enhanced effects. The antibody with its Y-shaped structure of 14.5 × 8.5 × 4.0 nm is about 20 times larger in volume than that of the DNA ligand. The antibody also has two binding sites at the ends of the Fab domains.

Considering the dimensions of the antibody and the nanopore lumen, it was assumed that only one of the Fab arms of an antibody can reach into the lumen of the nanopore. This is in contrast to conventional kinetic studies of antibody binding on ligand-coated surfaces where both single Fab and double Fab interaction occurs, often without differentiating between the two processes.

For our analysis of antibody binding, we utilized an antibiotin antibody with a reported *K*_d_ for binding to biotin of 1.7 × 10^–9^ M (refs 94 and 111). The biotin tag used in our study was installed at the nanopore entrance (NP-O^Biot^) or inside the pore lumen (NP-I^Biot^) (Figure 3A). These pores were fabricated from DNA nanopores NP-O^DNA^ and NP-I^DNA^ that carry the 20 nt single-stranded DNA by hybridizing a terminally biotin-tagged oligonucleotide of complementary sequence for either 1 or 3 h (Figure S12). Initial experiments with assembled NP-O^Biot^ and NP-I^Biot^ revealed that maximum binding of the antibody L^Ab^ was achieved within less than 5 min (Figure S16). As these binding kinetics are too fast to directly determine *k*_on_ with the used dc-FCCS setup, we chose to first measure the equillibrium dissociaton constant *K*_d_ and then obtain *k*_off_ to calculate *k*_on_ using eq 5.

**Figure 3:**
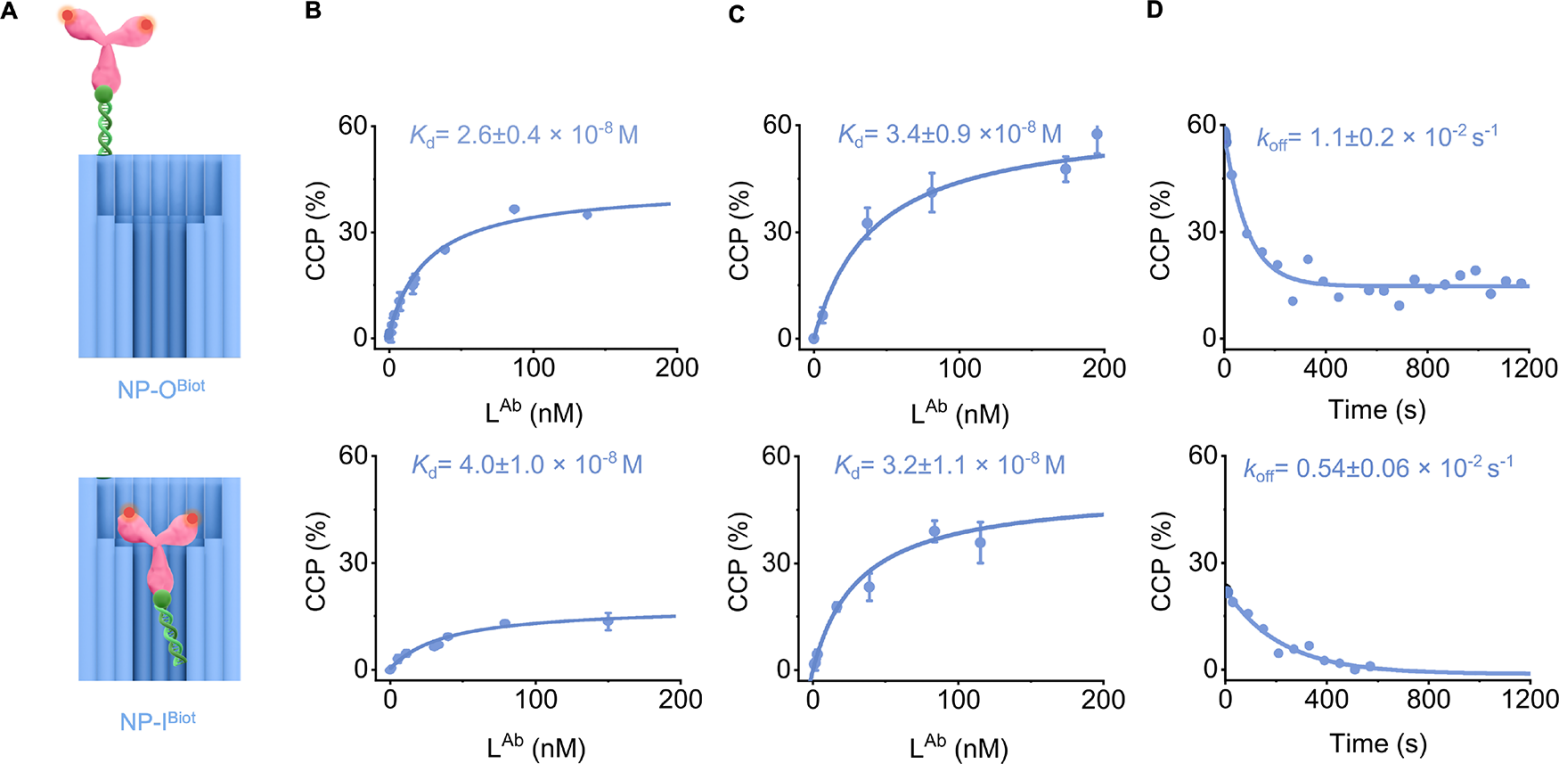
Binding affinities and dissociation rate constants of antibody–biotin interaction within and out of nanoconfinement. (A) Schematic drawing on the interaction of L^Ab^ with NP-O^Biot^ (top) and NP-I^Biot^ (bottom). (B–D) Data for NP-O^Biot^ (top) and NP-I^Biot^ (bottom). (B) Plot on the formation of the NP·L complexes, expressed as CCP, as a function of L^Ab^ concentration. NP-O^Biot^ and NP-I^Biot^ were formed from NP-O^DNA^ and NP-I^DNA^ by incubation with the biotin-tagged oligonucleotide at 1.3 × excess for 1 h prior to L^Ab^ addition. For NP-O^DNA^, the L^DNA^ concentrations ranged from 1.1 to 140 nM, and for NP-I^DNA^ from 1.1 to 150 nM. (C) Same as (B), but NP-O^Biot^ and NP-I^Biot^ were formed by incubation with the biotin-tagged oligonucleotide at 3 × excess for 3 h prior to L^Ab^ addition. (D) Kinetic traces showing the decay of preformed complexes between fluorophore-labeled NP-O^Biot^ and NP-I^Biot^ and L^Ab^ (250 and 30 nM, respectively) after the addition of an unlabeled antibody (1000 nM) following a decay constant *k*_off_.

To determine *K*_d_ for antibody binding to the biotinylated nanopores, we added varying concentrations of L^Ab^ to NP-O^Biot^ or NP-I^Biot^ and measured the binding degree of CCP via dc-FCCS. These NP-O^Biot^ or NP-I^Biot^ pores had been obtained from 1 h of incubation of NP-O^DNA^ and NP-I^DNA^ with a biotinylated DNA strand in 1.3 × molar excess. Plotting the resulting CCP for formation of the nanopore-ligand complex against the L^Ab^ concentration yielded binding profiles (Figure 3B), which were then fitted with eq 4 to yield *K*_d_. The values were 2.6 ± 0.4 × 10^–8^ M for NP-O^Biot^ and 4.0 ± 1.0 × 10^–8^ M for NP-I^Biot^, which is within error. Very similar *K*_d_ values of 3.4 ± 0.9 × 10^–8^ and 3.2 ± 1.1 × 10^–8^ M were obtained for binding to NP-O^Biot^ and NP-I^Biot^, respectively, which had been obtained after 3 h of incubation with the biotinylated DNA strand (Figure 3C and Table 1). The absolute CCP values for the pores from 1 h of biotin-oligo incubation (Figure 3B) were lower than for the pores from 3 h of incubation (Figure 3C). This is in line with the results from DNA–DNA hybridization (Figure 2) where longer incubation also leads to a higher binding extent.

The similarity of the four *K*_d_ values for antibody–nanopore binding illustrates three important points. First, a full loading of all nanopores with the biotin tag is not required to obtain meaningful *K*_d_ values. Second, the similar *K*_d_ values for NP-I^Biot^ and NP-O^Biot^ likely reflect that nanoconfinement for the antibody–biotin interaction leads to compensatory effects for association and dissociation kinetics, as was initially observed for the DNA–DNA interaction in the nanopore. Finally, our *K*_d_ values in the low 10^–8^ M range are similar to literature values for Fab fragment binding in bulk^112^—in line with our monovalent binding—but different from the 10^–9^ M range for full antibody binding,^111–113^ which may involve bivalent binding and rebinding.

After having measured the equilibrium dissociation constant *K*_d_ for the antibody–biotin interaction, we next determined the dissociation rate constant *k*_off_. As previously pointed out, acquiring direct kinetic binding curves was not possible as the antibody–biotin interaction occurred too fast to be kinetically resolved with our dc-FCCS setup. Consequently, we determined *k*_off_ using a competition experiment where a nonfluorescent antibody was added to a premixed complex between NP-I^Biot^ or NP-O^Biot^ and fluorophore-labeled L^Ab^.

The displacement of biotin-bound fluorophore-labeled L^Ab^ by the nonlabeled antibody was expected to follow dissociation kinetics with the rate constant *k*_off_. The displacement kinetics were determined by tracking the decrease in CCP over time after the addition of an unlabeled antibody (Figure 3D). The decay curve was fitted with

$$\mathrm{CCP}=A^{\prime }\cdot \exp^{-k_{\mathrm{off}}\cdot t}\;+o$$ 6

where *A*′ represents the percentage of CCP at the start of the competition reaction and *o* is the offset from zero for incomplete displacement of the labeled antibody. The resulting *k*_off_ values were 1.1 ± 0.2 × 10^–2^ s^–1^ for NP-O^Biot^ and 0.54 ± 0.06 × 10^–2^ s^–1^ for NP-I^Biot^ (Table 1). Using the measured *K*_d_ and *k*_off_ values, we then calculated *k*_on_ for both NP variants (Table 1). The values of 3.3 ± 1.1 × 10^5^ and 1.7 ± 0.6 × 10^5^ M^–1^ s^–1^ for NP-O^Biot^ and NP-I^Biot^, respectively, indicate slower kinetics for the association of the antibody to the recessed biotin tag. The kinetic and affinity characteristics of antibody binding are similar to those of DNA hybridization. For protein and DNA, slower association and slower dissociation under nanoconfinement compensate for each other to yield the same affinities for confined versus nonconfining conditions. The confinement effect on kinetics may be similar for antibody and DNA as the antibody’s Fab unit has approximately the same cross-sectional width as the extended DNA strand upon binding to the DNA receptor strand.

### Streptavidin Binding to Biotinylated Nanopores

We finally used dc-FCCS and DNA nanopores to probe the molecular interaction between streptavidin and biotin which has one of the highest affinities with *K*_d_ values of 10^–12^–10^–14^ M.^114–117^ As streptavidin with a diameter of about 5.5 nm is 4 times smaller than an antibody and has four binding sites, we wondered whether nanoconfinement effects would also take place for the smaller multivalent protein. We were also curious whether tight binding for streptavidin could be resolved by our experimental approach. To answer these questions, we used nanopores carrying a biotin tag at the pore entrance (NP-O^Biot^) or within the pore lumen (NP-I^Biot^). The nanopores were obtained as described before by incubating the corresponding DNA-nanopores for 1 h with a 1.3-fold excess of the biotinylated oligonucleotide.

We determined *k*_on_ for the biotin–streptavidin interaction following the same route as that used for the antibody. L^Strep^ was added to NP-O^Biot^ and NP-I^Biot^ (Figure 4A), and dc-FCCS tracked the formation of the binary complex via CCP as a function of incubation time and for different L^Strep^ concentrations (Figure 4B). The resulting *k*_eq_ values were then plotted against L^Strep^ concentration (Figure 4C), and the curves were fitted to obtain the association rate constant *k*_on_. The values of *k*_on_ were similar for NP-O^Biot^ and NP-I^Biot^ with 1.2 ± 0.1 × 10^6^ and 1.0 ± 0.2 × 10^6^ M^–1^ s^–1^, respectively (Table 1). While the values agree with those in the literature,^118,119^ their similarity suggests no nanoconfinement effect for the streptavidin protein. A plausible explanation is that streptavidin, with its four binding sites for biotin, has a higher chance of binding biotin without requiring major reorientation within the confinement, something which would automatically lower any kinetic confinement effect.

**Figure 4:**
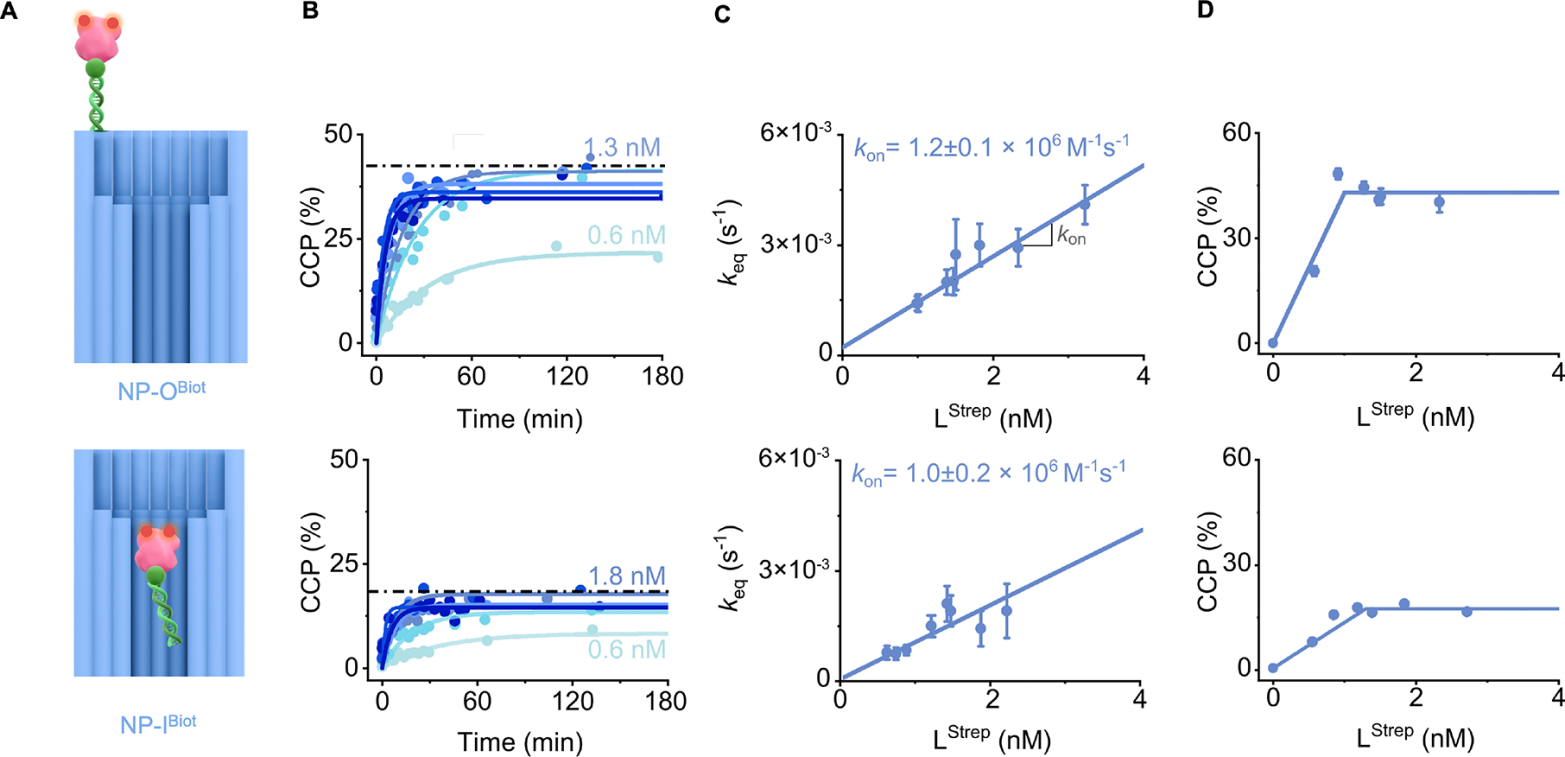
Binding affinities and binding kinetics of streptavidin binding to biotin within and out of nanoconfinement. (A) Schematic drawing on the interaction of L^Strep^ with NP-O^Biot^ (top) and NP-I^Biot^ (bottom). (B–D) Data for NP-O^Biot^ (top) and NP-I^Biot^ (bottom). (B) Kinetic traces for the binding of the NP variants to L^Strep^, plotted as CCP as a function of incubation time at different L^Strep^ concentrations. (C) Plots of equilibrium rate constant *k*_eq_—obtained from the exponential fits to graphs in (B)—versus L^Strep^ concentration to obtain *k*_on_ from the slope of the linear fit. (D) Plots of CCP—after 180 min equilibration of binding—versus L^Strep^ concentration to obtain *K*_d_. For NP-O^Biot^, L^Strep^ concentrations were 0.6, 0.9, 1.3, 1.5, 1.5, and 2.3 nM, and for NP-I^Biot^, the concentrations were 0.6, 0.7, 0.8, 1.2, 1.4, 1.8, and 2.7 nM. The concentration of the nanopores was 1.3 nM.

After establishing *k*_on_, we determined *K*_d_ for the streptavidin–biotin interaction. The degree of binding of CCP was obtained as a function of L^Strep^ concentration after 3 h of incubation (Figure 4D). The binding profile was very steep with full binding at a very low L^Strep^ concentration of 1 nM, in line with the well-known high affinity. Lower L^Strep^ concentrations could not be used given the limiting sensitivity of dc-FCCS. The traditional Langmuir–Hill fitting for the binding profiles (Figure 4D) was not possible and was attempted with another fit (eq S19) to yield *K*_d_ values in the low pM range with unavoidably higher errors (Table 1), indicating that the affinities for this interaction are difficult to resolve with fluorescence cross-correlation spectroscopy.

## Conclusions

Considering the importance of molecular interaction kinetics for understanding biology and enhancing biomedicine and biotechnology, this study set out to achieve one primary aim: To characterize the influence of nanoconfinement on molecular interaction kinetics and thereby address contradictions and questions on whether confinement should occur for binding with rates below the diffusion limit. This primary aim should be conducted with a single receptor to minimize rebinding and avoid the upconcentration of ligands in nanoconfinement. A second aim was to pioneer DNA nanopores as a model platform to study confinements given DNA nanotechnology’s ease of installing a single receptor site inside or outside a confining lumen as well as the simple choice of receptors to bind representative, diffently sized biomolecular ligands. A third aim was to establish dc-FCCS as a powerful single-molecule alternative to classical force and electrical single-molecule methods, which suffer from shortcomings due to sample surface immobilization and low throughput.

The research outcomes of our study can be summarized in four highlights. First, fluorescence cross-correlation spectroscopy and DNA nanopores were successful in providing easy-to-access, high-data-number, reliable kinetic analysis of molecular interaction in and out of nanoconfinement. The analysis recorded thousands of single molecules per second and revealed small kinetic differences as shown by the 2- to 3-fold higher or lower kinetic rate constants, which is considerable when compared to typical errors of other techniques.^41,120,121^ In addition, the approach detected kinetic rate constants ranging from 10^5^ to 10^6^ M^–1^ s^–1^ for *k*_on_ and from 10^–2^ to 10^–4^ s^–1^ for *k*_off_. Similarly, the affinities were measured with *K*_d_ values from 10^–8^ M to 10^–12^ M even though the latter extreme affinity is beyond the lower boundary of the typical sensitivity range of FCCS from 0.5 to 100 × 10^–9^ M. As FCCS measures the codiffusion of the ligand and receptor, it provides unequivocal confirmation of successful binding, which is more challenging to obtain with single-molecule fluorescence resonance energy transfer, as the FRET signal could reflect nanoscale proximity between two FRET fluorophores without binding.^122^ FCCS does not directly provide the same access to spatial information about the geometry of the molecular interaction as atomic force microscopy and offers less detailed insight into the interaction when compared to single-channel current recordings, as indicated by the power of DNA nanopore sequencing. Yet, within the given boundaries, our study has found new important insights.

As second highlight, our study provides evidence that nanoconfinement influences biomolecular binding kinetics by using an experimental system operating outside the previously tested parameter space Intuitively, placing the receptor in a confined space might act like a bottleneck, reducing both the association and dissociation rates proportionally. However, theoretical models predict no significant confinement effects when association rates are well below the diffusion limit.^29–31^ Furthemore, published data have shown increased association rates due to the effective up-concentration of ligands in small enclosed volumes,^32,36,37^ or changes in *K*_d_ under confinement.^33,35,37^ Our report is different by using molecular interaction pairs with association kinetics 2 orders of magnitude slower than the diffusion limit. In addition, we use as confining environment a nanopore with two defined openings which enable ligand diffusion into and out of the confining volume. This is different to previously published small enclosed nanoconfinements where ligand entry and exit cannot happen as readily.^40^

In contrast to theoretical predictions and previous findings, our experiments show that nanoconfinement clearly reduced association and dissociation kinetics by approximately 3-fold compared with free access. This is plausible, as nanoconfinement decreases the translational freedom of ligand movement to and away from the receptor site. Moreover, we unequivocally determined the kinetics for monovalent binding for bivalent antibodies and tetravalent streptavidin, which is often a challenge for conventional atomic force microscopy and surface plasmon resonance, where multibinding cannot be excluded even for strongly diluted conditions.^67,123,124^ The observed confinement effect for DNA–DNA hybridization and antibody–biotin binding can be explained by conformational-entropic penalties during binding. Long molecules like DNA face greater restrictions in confined spaces, making it harder for them to properly orient for hybridization. The situation is different with streptavidin, which is compact and has multiple symmetrically arranged binding sites, which likely reduces such penalties. This hypothesis on compensatory entropic penalties can be tested in future experiments by separately assessing entropy and multivalency effects on *k*_on_ and *k*_off_.

As the third highlight of our study, the kinetic slow-down effects compensate for each other to yield the same overall affinity compared to nonconfined receptors. This has previously not been observed and demonstrates that measuring solely affinity can mask nanoconfinement effects. This finding can change our understanding of many nanoconfined systems in biology and biotechnology.

Finally, our methodological approach for kinetic analysis of binding lays out a path for future extension to other DNA nanostructures as well as biological channels, something that is only beginning to be explored. To realize the potential for acquiring high data numbers from water-soluble ligand receptor complexes, membrane channels could be placed in water-soluble membrane nanodiscs. The required fluorophore tagging of both the ligand and biological channel is feasible, considering the wide range of tagging protocols. As molecular species can be detected in concentrations ranging from 0.5 to 100 nM, the study of most binding kinetics with associated affinities down to the low nM range would be accessible.

In conclusion, our study establishes that nanoconfinement affects molecular interaction at association rates even below the diffusion limit, implements a new combinatory approach for single-molecule analysis, and can serve as inspiration for kinetic molecular analysis of other DNA nanostructures and biological channels of biotechnological and biomedical interest.

## Supplementary Material



## Notes

Detailed experimental methods, table of DNA sequences, additional fluorescence correlation analysis (PDF)
