# Supplementary material for: Molecular Interactions within Nanoconfinement of Model DNA Nanostructures Controlled by Compensatory Kinetics as Revealed by Single-Molecule Fluorescence Analysis
Source: JACS Au. 2025 Aug 28;5(9):4427–38. doi: 10.1021/jacsau.5c00774 (PMC12458038; doi:10.1021/jacsau.5c00774)
Supplement: Supplementary file 1 [file au5c00774_si_001.pdf]

Supplementary Information

Molecular Interactions within Nanoconfinement of Model DNA Nanostructures Controlled by Compensatory Kinetics as Revealed by Single-Molecule Fluorescence Analysis

Nora Hagleitner-Ertuğrul[a], Yongzheng Xing[b], Juergen Pfeffermann[a], Alexia Rottensteiner[c], Anna Gaugutz[d], Denis G. Knyazev[a], Peter Pohl[a], Stefan Howorka[c,*]

[a] *Institute of Molecular Biophysics and Membrane Biophysics, Johannes Kepler University Linz, Gruberstraße 40, 4020 Linz, Austria*

[b] *National Engineering Research Center for Colloidal Materials, School of Chemistry and Chemical Engineering, Shandong University, 27 Shanda South Road, Jinan 250100, China*

[c] *Department of Chemistry, Institute of Structural Molecular Biology, University College London, London WC1H 0AJ, United Kingdom*

[d] *Institute of Applied Physics, TU Wien, Wiedner Hauptstraße 8-10, 1040 Vienna, Austria*

Table of Contents

1. Materials and Methods 1

1.1. Materials 3

1.2. Nanopore assembly 3

1.3. Agarose gel electrophoresis 3

1.4. Purification 3

1.5. Transmission electron microscopy 3

1.6. Characterization of nanopore variants and ligands L^{DNA}, L^{Strep}, and L^{Ab} using fluorescence correlation spectroscopy 4

1.6.1. Determining optimal laser power for nanopore variants and ligands L^{DNA}, L^{Strep}, and L^{Ab} to avoid photobleaching and saturation 4

1.6.2. Calculation of diffusion coefficient and hydrodynamic radius of nanopore variants and ligands L^{DNA}, L^{Strep}, and L^{Ab} 4

1.7. Dual-color fluorescence cross-correlation spectroscopy 5

1.7.1. The general 1:1 binding case 5

1.7.2. Calibration of confocal volumes using a fluorescently double labeled DNA-duplex 6

1.7.3. Calculation of K_d, k_{on}, and k_{off} for DNA hybridization 6

1.7.4. Control experiments to exclude false-positive cross-correlation from ligand retention in the confined pore lumen 7

1.7.4.1. L^{DNA} addition to a nanopore without receptor 7

1.7.4.2. Probing NP-I^{DNA} with $L^{DNA,mis}$ containing 11 mismatches 7

1.7.5. Characterizing the binding extent of a biotin-tagged oligonucleotide to NP-O^{DNA} and NP-I^{DNA} 7

1.7.6. Determination of K_d, k_{on}, and k_{off} for the interaction of NP-I^{Biot} and NP-O^{Biot} with anti-biotin antibody 7

1.7.7. Determination of K_d and k_{on} for the interaction of NP-I^{Biot} and NP-O^{Biot} with streptavidin 8

2. Supplementary Results 9

3. Supplementary Tables 10

4. Supplementary Figures 18

1.Material and Methods

1.1. Materials

Unmodified and fluorophore-labeled DNA oligonucleotides were purchased from Integrated DNA Technologies (Leuven, Belgium) on a 25 nmole or 100 nmole scale with standard desalting, HPLC or PAGE purification. Biotinylated DNA oligonucleotides were acquired from Biomers.net (Ulm, Germany) on a 50 nmole scale with HPLC purification. The scaffold strand, M13mp18 type 7249, was obtained from Tilibit nanosystems (Frankfurt, Germany) at a concentration of 400 nM. Fluorescently labeled streptavidin was purchased from Biomol GmbH (Hamburg, Deutschland). Biotin monoclonal antibody was received from Jackson ImmunoResearch (Cambridge, United Kingdom). Free dyes, Rhodamine 6G and AF647 maleimide were procured from Merck (Darmstadt, Germany) and Jena Bioscience (Jena, Germany). All other reagents and solvents were purchased from Sigma-Aldrich unless stated otherwise.

1.2. Nanopore assembly

DNA nanopore NP-N lacks a DNA receptor, and DNA nanopores NP-O^{DNA} and NP-I^{DNA} carry a single stranded DNA receptor in outer and inner lumen position (Table S1). The 2D connectivity map of DNA nanopore NP-N is shown in Figure S1. The sequences of DNA oligonucleotides for the assembly of NP-N are in Table S4. The sequences of DNA oligonucleotides for the assembly of NP-O^{DNA} and NP-I^{DNA} carrying fluorophore tags are in Tables S4, S5, and S7. The nanopores were self-assembled in a one-pot reaction containing a mixture of m13mp18 scaffold (20 µL, 0.1 µM), 10 × excess of respective staples strands and 50 × excess of the AF647 carrying oligonucleotide (1 µL, 100 µM) and 0.5 × TAE buffer, supplemented with 16 mM $MgCl_2$ (10 µL), topped up with deionized H_2O to 100 µL. Thermal annealing of NP-O^{DNA} and NP-I^{DNA} was achieved with the Analytik Jena FlexCycler (Germany) by first heating up to 75 °C for 10 min and subsequently cooling from 65 °C to 25 °C (1 h per °C), and 25 °C to 10 °C (5 min per °C). The nanopores were stored at 4 °C until used.

1.3. Agarose gel electrophoresis

The DNA nanopores (100 µL, 20 nM) were mixed with gel loading buffer (10 µL; purple, no SDS; New England Biolabs) and loaded into the wells of a 1 % agarose gel, stained with GelRed (15 µL, 10000 × in water) in 0.5 × TAE, 10 mM $MgCl_2$ buffer. A 1000 base-pair (bp) marker (ThermoScientifc, UK) was used as the reference standard. The gel was run at 65 V for 90 min in an ice bath. DNA bands were visualized by ultraviolet illumination. After electrophoresis, the gel bands were imaged with a gel imaging system (ChemiDoc MP imaging system, BioRad) and analyzed with the image lab software (Homepage). The usage of red filter sets allowed the visualization of fluorescently labeled NP variants.

1.4. Purification

After folding and electrophoresis, bands corresponding to NP-O^{DNA} and NP-I^{DNA} were identified and neatly cut out of the gel with a scalpel. The bands were transferred into separate 1.5 mL Eppendorf tubes and stored at -20 °C for 30 min. Subsequently, the bands were further cut into smaller pieces and filtered through “Freeze ‘N Squeeze DNA extraction spin columns” (Bio-Rad Laboratories Incorporation) for 3 min at 13,000 × g and 4 °C. The filtered fraction containing purified NPs was stored at 4 °C for further usage.

1.5. Transmission electron microscopy

Purified NP-N (6 µL, 5 nM) was added onto glow discharge-treated TEM grids and stained with 0.5 % uranyl acetate solution. TEM analysis was performed on a JEM-2100 electron microscope (JEOL) operated at 200 kV and images were acquired with an Orius SC200 camera.

1.6. Characterization of nanopore variants and ligands L^{DNA}, L^{Strep}, and L^{Ab} using fluorescence correlation spectroscopy

Fluorescence correlation spectroscopy (FCS) analysis used a commercial laser scanning microscope (ConfoCor 3, Carl Zeiss, Jena, Germany) equipped with a 40× water immersion objective. Measurements were performed using a 635 nm secondary dichroic mirror with a 650 nm longpass filter in the red channel and a 505 - 540 nm bandpass filter in the green channel. 30 µL of sample (1 nM to 100 nM) was pipetted into a well glued to the cover glass. The measurement time was set to 20 sec and an average of 3 measurements were taken.

1.6.1. Determining optimal laser power for nanopore variants and ligands L^{DNA}, L^{Strep}, and L^{Ab} to avoid photobleaching and saturation

40 µL of the freshly purified nanopores (4.5 nM) was measured by varying the laser power (Helaser, 5 mW) between 0 µW to 100 µW, and the autocorrelation curves were measured yielding the increase in molecular brightness (counts per molecule, CPM, in kHz) with laser power. For analysis of ligands, stocks of L^{DNA} (100 µM), L^{Strep} (100 nM) and L^{Ab} (0.5 mg/ml) were diluted to a final concentration of 30-50 nM using 0.5 × TAE supplemented with 10 mM $MgCl_2$. The laser power (488 nm diode, 1 mW) was varied between 0 to 660 µW to measure the dependence of molecular brightness on laser power.

1.6.2. Calculation of diffusion coefficient and hydrodynamic radius of nanopore variants and ligands L^{DNA}, L^{Strep}, and L^{Ab}

The diffusion time of nanopores, L^{DNA}, L^{Strep} and L^{Ab}, was measured via FCS as mentioned in section 1.6. The diffusion time was obtained from the fit of the respective autocorrelation functions $G(\tau)$ by either the one-component model for translational diffusion[1,2]

$$G_1(\tau) = \frac{1}{N \cdot \left(1 + \frac{\tau}{\tau_D}\right)} + 1 \tag{1}$$

For AF647 labeled NPs an additional triplet term has been added to equation 1:

$$G_{1,T} = \frac{1}{N \cdot \left(1 + \frac{\tau}{\tau_{D,1}}\right)} \cdot \left(1 + \frac{T}{1-T} \cdot e^{-\frac{\tau}{\tau_{triplet}}}\right) + 1 \tag{2}$$

where T is related to the dark fraction of the respective dye. The diffusion coefficients of NPs and ligands were calculated using the following formula:[2]

$$D = \frac{\omega^2}{4 \cdot \tau_D} \tag{3}$$

where τ_D represents the characteristic diffusion time of NPs in the red channel or ligands (L^{DNA}, L^{Strep} and L^{Ab}) in the green channel and ω is the lateral radius of either the red or green confocal volume. The lateral radii[2], ω_g and ω_r, were obtained from calibration experiments using labels

with known diffusion coefficients *(D)*, Rhodamine 6G (Merck, Germany) and Alexa Fluor 647 maleimide (Jena Bioscience, Germany).

Based on the evaluated diffusion coefficient, the hydrodynamic radius of each species was determined based on the Stokes-Einstein equation:[3]

$$r_{\mathrm{H}} = \frac{k_{\mathrm{B}} \cdot T}{6 \cdot \pi \cdot D \cdot \eta} \tag{4}$$

where k_{B} is the Boltzmann constant, T is the temperature and η is the viscosity of the solvent.

1.7. Dual-color fluorescence cross-correlation spectroscopy

Cross-correlation measurements were performed using a 635 nm secondary dichroic mirror with a 505-540 nm bandpass filter in the green channel, and a 650 nm longpass filter in the red channel (Figure S11). Based on the photobleaching threshold determined for all nanopores NP-O^{DNA}, NP-I^{DNA}, NP-O^{Biot}, and NP-I^{Biot} in the red channel, and the ligands L^{DNA}, L^{Strep}, L^{Ab} (Tables S2, S3) in the green channel, the laser power was furthermore adjusted such that the brightness ratio of red to green signal was roughly 3:1 to avoid bleed-through.

1.7.1. The general 1:1 binding case

The obtained fluorescence intensity traces detected in the green and red detection channels were analyzed by the correlation function[3]

$$G(\tau) = \frac{\langle F(t) \cdot F(t+\tau) \rangle}{\langle F(t) \rangle^2} \tag{5}$$

whereby G(τ) is the autocorrelation function, and F(t) and F(t + τ) are fluorescence intensities at time t and time t + τ, respectively; τ is the lag time and the angle brackets symbolize averaging over all t. G(τ) is high for τ approaching zero, and decreases with increasing τ, as shown for L and NP (Figure 1D, right panel, red and blue graph). G(τ) is a measure of the self-similarity of the fluorescence signal and yields characteristic temporal parameters such as the diffusion time of the detected species.[2-3]

To quantify the co-occurrence of signals for NP and L from both detectors corresponding to NP●L, the cross-correlation curves of both fluorescent intensities were obtained with[3]

$$G_{NP \bullet L}(\tau) = \frac{\langle F_{NP}(t) \cdot F_L(t+\tau) \rangle}{\langle F_{NP}(t) \rangle \langle F_L(t) \rangle} \tag{6}$$

where F_{NP} and F_L are the fluorescence intensities in the NP and L fluorescence detection windows, respectively. The corresponding graph for cross-correlation is shown in Figure 1D (black curve).

In the case of 1:1 stoichiometric binding, the percentage of NP and ligand bound, CCP and CCP_L, can be calculated from the following ratios of cross-correlation ($G_{\mathrm{NP \bullet L}}$) and autocorrelation amplitudes (G_{NP} and G_{L}):[4]

$$CCP = \frac{G_{\mathrm{NP \bullet L}}}{G_{\mathrm{L}}} \cdot 100 \tag{7}$$

$$CCP_{\mathrm{L}} = \frac{G_{\mathrm{NP\bullet L}}}{G_{\mathrm{NP}}} \cdot 100 \tag{8}$$

The number of particles detected was obtained from the fit of the respective autocorrelation (for NPs and ligands) and cross-correlation (for NP•L) functions $G(\tau)$ by the one- (equation 1) or two-component model for translational diffusion:[2]

$$G_2(\tau) = \frac{x_1}{N \cdot \left(1 + \frac{\tau}{\tau_{\mathrm{D},1}}\right)} + \frac{x_2}{N \cdot \left(1 + \frac{\tau}{\tau_{\mathrm{D},2}}\right)} + 1 \tag{9}$$

where $\tau_{D,1}$ and $\tau_{D,2}$ represents the diffusion time through the confocal volume. x_1 and x_2, denote the fractional amplitudes of freely diffusing and bound ligand. The cross-correlation percentage, *CCP* and CCP_{L}, was corrected by accounting for the difference in size of the red and green confocal volumes by using the following formula:[4]

$$CCP_{\mathrm{v}} = CCP \cdot \frac{V_{\mathrm{eff}}}{V_{\mathrm{g}}} \tag{10}$$

$$CCP_{\mathrm{L,v}} = CCP_{\mathrm{L}} \cdot \frac{V_{eff}}{V_{\mathrm{r}}} \tag{11}$$

where the effective cross-correlation volume (V_{eff}) is defined as[4,5]:

$$V_{\mathrm{eff}} = \pi^{\frac{3}{2}} \cdot \left(\frac{\omega_{\mathrm{r}}^2 + \omega_{\mathrm{g}}^2}{2}\right) \cdot S \tag{12}$$

where S is a structural parameter, which was set equal for both channels, $S_{\mathrm{g}} = S_{\mathrm{r}} = 6$, and ω is the respective lateral radius of the confocal volume, ω_{g} or ω_{r}, of the green or red confocal volume. Confocal volumes, V, in the red and green channels, were calculated as:[2]

$$V = \pi^{\frac{3}{2}} \cdot \omega^2 \cdot S \tag{13}$$

1.7.2. Calibration of confocal volumes using a fluorescently double labeled DNA duplex

To determine the degree of overlap of the red and green confocal volume, which determines the maximum percentage of receptor and ligand-bound that can be obtained in our FCS setup, we used a dsDNA labeled with two fluorophores, Alexa Fluor 488 and Cy5 (Table S8). Measurements were conducted as section 1.7. and calculations were performed as in section 1.7.1.

1.7.3. Calculation of K_{d}, k_{on}, and k_{off} for DNA hybridization

To measure k_{on}, different reaction mixtures of the NP (NP-O^{DNA}, NP-I^{DNA}) and its ligand, L^{DNA}, were prepared in 0.5 × TAE with 10 mM $MgCl_2$ up to a final volume of 60 µL. In all reaction mixtures, the concentration of the NP was kept constant at 1 to 1.5 nM, while the concentration of L^{DNA} was varied. The samples were incubated at RT and measured at different time points ranging from 0 min to 180 min using dc-FCCS.

For all reaction mixtures, the change in percentage NP bound, CCP, during the set timeframe (0 - 180 min) was fitted with the following equation to obtain k_{eq} (s^{-1}):[6]

$$CCP\ (\%) = a \cdot \left(1 - e^{-k_{eq} \cdot t}\right) \tag{14}$$

The k_{eq} constant of each reaction mixture was then plotted versus the concentration of L^{DNA} in the reaction mixture. Fitting the resulting curves with the following linear fit yields k_{on} of the respective receptor-ligand interaction.[6]

$$k_{eq} = k_{on} \cdot [L] + k_{off} \tag{15}$$

K_d was evaluated taking CCP at different ligand concentrations and incubation time of 3 h and plotting it versus L^{DNA} concentration. Resulting data plot was fitted with the following fit:[6]

$$CCP\ (\%) = A \cdot \frac{[L]}{[L] + K_d} \tag{16}$$

The association rate constant, k_{on} was then calculated using measured K_d and k_{off} values using following equation:[6]

$$k_{on} = \frac{k_{off}}{K_d} \tag{17}$$

1.7.4. Control experiments to exclude false-positive cross-correlation from ligand retention in the confined pore lumen

1.7.4.1. L^{DNA} addition to nanopore without receptor

A nanopore lacking the receptor site (NP) was assembled and purified as described in sections 1.2–1.4. Different reaction mixtures of NP and the ligand, L^{DNA}, were prepared in 0.5 × TAE with 10 mM $MgCl_2$ up to a final volume of 30 µL. In all reaction mixtures, the concentration of the NP was kept constant at 10 nM, while the concentration of L^{DNA} was varied (2nM – 80nM). The samples were incubated for 1 h at RT and binding was measured using dc-FCCS as described in section 1.7 (Figure S13).

1.7.4.2. Probing NP-I^{DNA} with $L^{DNA,mis}$ containing 11 mismatches

NP-I^{DNA} was assembled and purified as described in Sections 1.2–1.4. Reaction mixtures containing NP-I^{DNA} and the mismatched ligand, $L^{DNA,mis}$, were prepared in 0.5× TAE with 10 mM $MgCl_2$ to a final volume of 30 µL. The concentration of NP-I^{DNA} was kept constant at 1–1.5 nM, while the concentration of $L^{DNA,mis}$ was varied between 1 nM and 64 nM. Samples were incubated for 3 hours at room temperature, and binding was assessed using dc-FCCS as described in Section 1.7 (Figure S14). Furthermore, NUPACK analysis of DNA duplex formation for fully complementary L^{DNA} and L^{DNA} containing 11 mismatches was performed (Figure S15).

1.7.5. Characterizing the binding extent of a biotin-tagged oligonucleotide to NP-O^{DNA} and NP-I^{DNA}

The extent of binding of biotin-tagged oligonucleotide to DNA-modified nanopores was determined by a set of two experiments (Figure S12). First, purified NP-O^{DNA} and NP-I^{DNA} were mixed with L^{DNA} at NP:L^{DNA} ratios of 1:0.5 or 1:3. NP-O^{DNA}:L^{DNA} was incubated for 1 h at RT and NP-I^{DNA}:L^{DNA} for 1 and 3 h at RT. The extent of NP•L formation expressed as *CCP* was determined via dc-FCCS. This experiment aimed to determine the maximum loading capacity for binding a fluorophore-labeled DNA strand under the set conditions. In the second

experiment, biotin-tagged oligonucleotide was used instead of L^{DNA}. NP-O^{DNA} and NP-I^{DNA} were incubated for 1 and 3 h with biotin tagged oligonucleotide, respectively. To determine the extent of biotinylated NPs, L^{DNA} was added in 3 x molar excess and incubated for 1 h for NP-O^{Biot} and 3 h for NP-I^{Biot} at RT. The *CCP* was determined via dc-FCCS, giving a measure for the unbiotinylated receptors. Lower *CCP* values indicate higher fractions of NP bound to biotin tagged oligonucleotide. In all reaction mixtures, the concentration of NPs was kept at 2 nM.

1.7.6. Determination of K_d, k_{on}, and k_{off} for the interaction of NP-I^{Biot} and NP-O^{Biot} with anti-biotin antibody

NP-O^{DNA} and NP-I^{DNA} were either incubated with 1.3 × molar excess biotin-tagged oligonucleotide for 1 h or 3 × molar excess of biotin tagged oligonucleotide for 3 h at RT. Different reaction mixtures of biotinylated NP (NP-O^{Biot}, NP-I^{Biot}) and L^{Ab}, were prepared in 10 mM $MgCl_2$ + 0.5 × TAE, with a final volume of 60 µL and incubated at RT for 15 min. In all reaction mixtures, the concentration of NPs was kept constant (2 nM, Figure S16), whereas L^{Ab} concentration was varied between 1 nM to 250 nM. Plotting CCP versus L^{Ab} concentration of each reaction mixture after 15 min yielded K_d via fitting with equation 14.

For both NPs (NP-O^{Biot} and NP-I^{Biot}), k_{off} was determined via a competition experiment, using unlabeled anti-biotin antibody. For NP-O^{Biot}, the reaction mixture containing 250 nM of L^{Ab} was mixed with unlabeled antibody (8.25 µL, 6.7 µM) to reach a final concentration of 1 µM unlabeled antibody. The decrease in CCP over time was measured. As the resulting ratio of labeled L^{Ab} and unlabeled antibody was 4, the resulting data plot was fitted using the following equation:[6]

$$CCP\ (\%) = A \cdot e^{-k_{\text{off}} \cdot t} + o \tag{18}$$

where *A* is the maximum amount of NP bound by L^{Ab}, before addition of unlabeled antibody and *o* represents the offset from 0 at the end of the reaction, which depends on the final ratio of L^{Ab}: unlabeled antibody. For NP-I^{Biot}, the k_{off} was measured starting from a reaction mixture, with just 30 nM of L^{Ab} and addition of unlabeled antibody with a final concentration of 1 µM. Higher excess of unlabeled antibody led to *o* = 0.

1.7.7. Determination of K_d and k_{on} for the interaction of NP-I^{Biot} and NP-O^{Biot} with streptavidin

Prior to k_{on} measurements, freshly purified NPs (NP-O^{DNA} and NP-I^{DNA}) were incubated with 1.3 × molar excess of biotin tagged oligonucleotide to biotinylate receptor sites. Different reaction mixtures of the NP (NP-O^{Biot} and NP-I^{Biot}) and L^{Strep}, were prepared in 10 mM $MgCl_2$ + 0.5 × TAE, with a final volume of 60 µL. In all reaction mixtures, the concentration of the NPs was kept constant (1 nM to 1.5 nM), while the concentration of L^{Strep} was varied. The samples were incubated at RT and measured at different time points ranging from 0 min to 180 min using dc-FCCS. The evaluation of k_{on} was performed as described in section 1.7.3. For evaluating K_d, following equation was used:[6]

$$CCP\ (\%) = A \cdot \frac{([NP]_{\text{total}} + [L]_{\text{total}} + K_{\text{d}}) - \sqrt{([NP]_{\text{total}} + [L]_{\text{total}} + K_{\text{d}})^2 - 4 \cdot [NP]_{\text{total}} \cdot [L]_{\text{total}}}}{2 \cdot [NP]_{\text{total}}} \tag{19}$$

2. Supplementary Results

Assembly and structural characterization of the DNA nanopore

Nanopores NP-I^{DNA} and NP-O^{DNA} were self-assembled in DNA origami fashion. The origami assembly mix contained 7249 nt-long M13mp18 scaffold strand (Table S4) and 206 custom-designed staple strands (Tables S5, S6) present in 10 × molar excess, along with AF647-carrying oligonucleotide (Figure S2, Table S7) in 50 × molar excess. The strands were self-assembled by thermal heating and cooling. Assembly of the NPs was confirmed by agarose gel electrophoresis and DNA-staining with ethidium bromide. Successful assembly was indicated by a single concise gel band for NP-O^{DNA} (Figure S3B, lane 3) which migrated slower than the scaffold band (Figure S3B, lane 2) due to the larger size and more compact nanopore structure compared to the flexible scaffold. The gel data for NP-I^{DNA} (Figure S3E) gave very similar results. The two pores had the same gel migration, suggesting that the DNA receptor strands was too small to alter electrophoretic movement. The assembled DNA nanopores were purified from the quicker migrating excess staple strands and AF647-modified oligonucleotides) by cutting out the pores' gel band and extracting the DNA pores. Re-analysis of the purified pores by gel electrophoresis confirmed removal of excess oligonucleotides given the absence of the corresponding gel bands as shown after ethidium bromide-stain (Figure S3B, lane 6) or long-wavelength fluorescence illumination (Figure S3C). The fluorescence analysis also demonstrated successful labeling of both nanopores with AF647 dyes (Figure S3C,F).

The dimensions of the DNA nanopore were established with transmission electron microscopy (TEM) of negatively stained samples. Analysis of TEM images (Figure S4) yielded a nanopore height of 45.9 ± 2.9 nm and an external width of 22.6 ± 1.3 nm (n = 23) which is in excellent agreement with the designed dimensions of 46.0 nm and 22.5 nm and the values of 44.5 ± 3.2 nm and 23.1 ± 2.2 nm from CanDo simulations which additionally showed a pore of low thermal fluctuations (Figure S5). oxDNA simulations of the DNA nanopores confirmed that the nanopore lumen does not collapse under thermal fluctuations (Figures S6, S7) and showed similar nanopore dimensions (Figure S8). Complementary analysis with fluorescence correlation spectroscopy (Figure S9) confirmed successful labeling of the pore with the AF647 fluorophores and also provided a diffusion coefficient for the pore of $5.02 \pm 0.44 \times 10^{-8}$ cm^2 s^{-1} which translates via the Stokes-Einstein equation into a hydrodynamic diameter of 48.0 ± 4.6 nm. Fluorescence correlation spectroscopy also confirmed successful dye-labeling of ligands (Figure S10).

3. Supplementary Tables

Table S1. Acronyms for the DNA pore variants, including type and position of receptor sites.

ID	Receptor position	Type of receptor	Fluorescent tag
NP-N	None	-	-
NP-O^{DNA}	Out	ssDNA	AF647
NP-I^{DNA}	Lumen	ssDNA	AF647
NP-O^{Biot}	Out	Biotin	AF647
NP-I^{Biot}	Lumen	Biotin	AF647

Table S2. Acronyms of ligands used in the study.

Acronym	Ligand	Fluorescent tag
L^{DNA}	ssDNA	AF488
$L^{DNA, mis}$	ssDNA	AF488
L^{Ab}	Anti-biotin antibody	AF488
L^{Strep}	Streptavidin	AF488

Table S3. Sequences of fluorophore-tagged or biotin-tagged oligonucleotides used as DNA ligands or means to attach biotin to the nanopore by binding to the complementary ssDNA overhang of oligonucleotides in Table S6.

ID	Sequence 5' → 3'
L^{DNA}	/5Alexa488N/ACCTTCCTCCGCAATACTCC
$L^{DNA, mis}$	/5Alexa488N/ATATTAATTTTTAATATTAA
$ssDNA^{5'biotin}$	/5biotin-TEG/ACCTTCCTCCGCAATACTCC

Table S4. Sequence of the M13mp18 scaffold strand with a length of 7249 nucleotides.

Sequence 5' → 3'
CTGGACAGAATTACTTTACCTTTTGTCGGTACTTTATATTCTCTTATTACTGGCTCGAAAATGCC<br>TCTGCCTAAATTACATGTTGGCGTTGTTAAATATGGCGATTCTCAATTAAGCCCTACTGTTGAGC<br>GTTGGCTTTATACTGGTAAGAATTTGTATAACGCATATGATACTAAACAGGCTTTTTCTAGTAAT<br>TATGATTCCGGTGTTTATTCTTATTTAACGCCTTATTTATCACACGGTCGGTATTTCAAACCATTA<br>AATTTAGGTCAGAAGATGAAATTAACTAAAATATATTTGAAAAAGTTTTCTCGCGTTCTTTGTCTT

GCGATTGGATTTGCATCAGCATTTACATATAGTTATATAACCCAACCTAAGCCGGAGGTTAAAA
AGGTAGTCTCTCAGACCTATGATTTTGATAAATTCACTATTGACTCTTCTCAGCGTCTTAATCTA
AGCTATCGCTATGTTTTCAAGGATTCTAAGGGAAAATTAATTAATAGCGACGATTTACAGAAGCA
AGGTTATTCACTCACATATATTGATTTATGTACTGTTTCCATTAAAAAAGGTAATTCAAATGAAAT
TGTTAAATGTAATTAATTTTGTTTTCTTGATGTTTGTTTCATCATCTTCTTTTGCTCAGGTAATTGA
AATGAATAATTCGCCTCTGCGCGATTTTGTAACTTGGTATTCAAAGCAATCAGGCGAATCCGTT
ATTGTTTCTCCCGATGTAAAAGGTACTGTTACTGTATATTCATCTGACGTTAAACCTGAAAATCT
ACGCAATTTCTTTATTTCTGTTTTACGTGCAAATAATTTTGATATGGTAGGTTCTAACCCTTCCAT
TATTCAGAAGTATAATCCAAACAATCAGGATTATATTGATGAATTGCCATCATCTGATAATCAGG
AATATGATGATAATTCCGCTCCTTCTGGTGGTTTCTTTGTTCCGCAAAATGATAATGTTACTCAA
ACTTTTAAAATTAATAACGTTCGGGCAAAGGATTTAATACGAGTTGTCGAATTGTTTGTAAAGTC
TAATACTTCTAAATCCTCAAATGTATTATCTATTGACGGCTCTAATCTATTAGTTGTTAGTGCTCC
TAAAGATATTTTAGATAACCTTCCTCAATTCCTTTCAACTGTTGATTTGCCAACTGACCAGATATT
GATTGAGGGTTTGATATTTGAGGTTCAGCAAGGTGATGCTTTAGATTTTTCATTTGCTGCTGGC
TCTCAGCGTGGCACTGTTGCAGGCGGTGTTAATACTGACCGCCTCACCTCTGTTTTATCTTCTG
CTGGTGGTTCGTTCGGTATTTTTAATGGCGATGTTTTAGGGCTATCAGTTCGCGCATTAAAGAC
TAATAGCCATTCAAAAATATTGTCTGTGCCACGTATTCTTACGCTTTCAGGTCAGAAGGGTTCTA
TCTCTGTTGGCCAGAATGTCCCTTTTATTACTGGTCGTGTGACTGGTGAATCTGCCAATGTAAA
TAATCCATTTCAGACGATTGAGCGTCAAAATGTAGGTATTTCCATGAGCGTTTTTCCTGTTGCAA
TGGCTGGCGGTAATATTGTTCTGGATATTACCAGCAAGGCCGATAGTTTGAGTTCTTCTACTCA
GGCAAGTGATGTTATTACTAATCAAAGAAGTATTGCTACAACGGTTAATTTGCGTGATGGACAG
ACTCTTTTACTCGGTGGCCTCACTGATTATAAAAACACTTCTCAGGATTCTGGCGTACCGTTCC
TGTCTAAAATCCCTTTAATCGGCCTCCTGTTTAGCTCCCGCTCTGATTCTAACGAGGAAAGCAC
GTTATACGTGCTCGTCAAAGCAACCATAGTACGCGCCCTGTAGCGGCGCATTAAGCGCGGCG
GGTGTGGTGGTTACGCGCAGCGTGACCGCTACACTTGCCAGCGCCCTAGCGCCCGCTCCTTT
CGCTTTCTTCCCTTCCTTTCTCGCCACGTTCGCCGGCTTTCCCCGTCAAGCTCTAAATCGGGG
GCTCCCTTTAGGGTTCCGATTTAGTGCTTTACGGCACCTCGACCCCAAAAAACTTGATTTGGGT
GATGGTTCACGTAGTGGGCCATCGCCCTGATAGACGGTTTTTCGCCCTTTGACGTTGGAGTCC
ACGTTCTTTAATAGTGGACTCTTGTTCCAAACTGGAACAACACTCAACCCTATCTCGGGCTATT
CTTTTGATTTATAAGGGATTTTGCCGATTTCGGAACCACCATCAAACAGGATTTTCGCCTGCTG
GGGCAAACCAGCGTGGACCGCTTGCTGCAACTCTCTCAGGGCCAGGCGGTGAAGGGCAATCA
GCTGTTGCCCGTCTCACTGGTGAAAAGAAAAACCACCCTGGCGCCCAATACGCAAACCGCCTC
TCCCCGCGCGTTGGCCGATTCATTAATGCAGCTGGCACGACAGGTTTCCCGACTGGAAAGCG
GGCAGTGAGCGCAACGCAATTAATGTGAGTTAGCTCACTCATTAGGCACCCCAGGCTTTACAC
TTTATGCTTCCGGCTCGTATGTTGTGTGGAATTGTGAGCGGATAACAATTTCACACAGGAAACA
GCTATGACCATGATTACGAATTCGAGCTCGGTACCCGGGGATCCTCTAGAGTCGACCTGCAGG
CATGCAAGCTTGGCACTGGCCGTCGTTTTACAACGTCGTGACTGGGAAAACCCTGGCGTTACC
CAACTTAATCGCCTTGCAGCACATCCCCCTTTCGCCAGCTGGCGTAATAGCGAAGAGGCCCGC
ACCGATCGCCCTTCCCAACAGTTGCGCAGCCTGAATGGCGAATGGCGCTTTGCCTGGTTTCCG
GCACCAGAAGCGGTGCCGGAAAGCTGGCTGGAGTGCGATCTTCCTGAGGCCGATACTGTCGT
CGTCCCCTCAAACTGGCAGATGCACGGTTACGATGCGCCCATCTACACCAACGTGACCTATCC
CATTACGGTCAATCCGCCGTTTGTTCCCACGGAGAATCCGACGGGTTGTTACTCGCTCACATTT
AATGTTGATGAAAGCTGGCTACAGGAAGGCCAGACGCGAATTATTTTTGATGGCGTTCCTATTG
GTTAAAAAATGAGCTGATTTAACAAAAATTTAATGCGAATTTTAACAAAATATTAACGTTTACAAT
TTAAATATTTGCTTATACAATCTTCCTGTTTTTGGGGCTTTTCTGATTATCAACCGGGGTACATAT
GATTGACATGCTAGTTTTACGATTACCGTTCATCGATTCTCTTGTTTGCTCCAGACTCTCAGGCA
ATGACCTGATAGCCTTTGTAGATCTCTCAAAAATAGCTACCCTCTCCGGCATTAATTTATCAGCT
AGAACGGTTGAATATCATATTGATGGTGATTTGACTGTCTCCGGCCTTTCTCACCCTTTTGAATC
TTTACCTACACATTACTCAGGCATTGCATTTAAAATATATGAGGGTTCTAAAAATTTTTATCCTTG
CGTTGAAATAAAGGCTTCTCCCGCAAAAGTATTACAGGGTCATAATGTTTTTGGTACAACCGAT
TTAGCTTTATGCTCTGAGGCTTTATTGCTTAATTTTGCTAATTCTTTGCCTTGCCTGTATGATTTA
TTGGATGTT

Table S5. Names and sequences of the DNA oligonucleotides used as staple strands for the formation of NP-N and other DNA nanopores.

ID	Sequence 5' → 3'
FYX001	GTTAAATAGCTTTCAACCCTCGTTTATCGTAGG

FYX002	ACTTATCAGGTGGCTCATTAAATCACCGGGTGAGA
FYX003	TTAGTTAAAGGCGTTAAATA
FYX004	GATGAAGAACAGTAACAGTA
FYX005	ACCAGGCAGGTCAGACGATATTAAAGCTGAG
FYX006	TAAATTTTTGGGGTCGAGGTGAGTCCACCGC
FYX007	TAACAGCAGCCTAATTACCTAAAACAAAATTAATTAAAAT
FYX008	AGAACGACGATAACGAGGCATTTTTAACAATATTTTGTTAAAATCCCA
FYX009	GTCAGGATTCCAGTCGGGAAACACGTATAACGTAAGC
FYX010	GGGCGCTAGGGCGCTGAATGCGCCGCTACAGGTCAGAGCG
FYX011	AGCCATGAGTGAGCTAACTCCCCGCTTTAGAGAGTAAGCTTAATTCAT
FYX012	AATTAACCGTTGAACTCAAACTATCGGCCAGCCATT
FYX013	ATTAAACAATAAATTGCGTAGATTTTCAGAATAATG
FYX014	CGGGGCCGTAAATCTGACCTAAAGAACGCAGA
FYX015	CATCTCATTTGGGGTTCCGACAGGCGAAAATC
FYX016	AATCGGTGATGTGCTGCAAGGCATAAA
FYX017	ACGAAAGAGGCATCATGAGGAAGTTTCCGACAGCAT
FYX018	CCTAACAAAGCAGAAACACGATTAGGAACAGTGCC
FYX019	GAGGCAGATTCACCAGTCAAGAACAATCCAG
FYX020	TCACGAAATTATTGCATACAAATTCTTAAGCCTGT
FYX021	ATCAGTTCAGCTCAGGTCGATCGTAATCATGGTCATTACG
FYX022	ATATCCAAGAGTCTATAATCAGTGAGGCCAGCTTTCCTCATG
FYX023	AGCAGCGAAAATTAAACGGGTAAAATTACGAAGGCCCCAGCG
FYX024	TTTTCAGACTTCTGGTGAGGCTGCGCAACTGTTAAGG
FYX025	GAATATTCTACAAAGGCAGCATGTCACGATTTTAATC
FYX026	CCAGACGTTAGTAAATGAATCGTAACGA
FYX027	ATTTTTACAGGTCAGGAAGACGGTAATCGTAAA
FYX028	ATTCCCGGAAACCAGGCAAAAAAAATCTTACCGCGCAGATGAG
FYX029	ATTATGGCAACATATAAAAGATATGGTTTATCATA
FYX030	GAAATCCGCGACCTGCTTGAAAGAGGATAGCAAGCCCAATCTAC
FYX031	CCCAATTCTGCCCCAGAATCGGCAAAATCCCTATAGG
FYX032	CCCGCACGACCATAAGTCCTTAGAAACCCATT
FYX033	TGCGTTTGAGCGTATTGTTCACCGCCT
FYX034	TCAAAACTTTCATTAGCGGGGTTACAGA
FYX035	TTTCATGCGCAGAGGCGA
FYX036	TGCGTGATTGTTCTTTCCAGTATTTATCCC
FYX037	GGAATCATGTAGGGCTTAAT
FYX038	GGAAGAGCCAGCGAATCAAGTTTCTTTGAGGACTAAAAAG
FYX039	ATCACAGTCACGTTACGCC
FYX040	CGAGTAACAACCCGTCGCGCATCGTAACCGGCATC
FYX041	TATTTAACTAAAGTACCGAC
FYX042	AGCGCGTTGTAATCAGGTTT
FYX043	GACAATCAGGCGTTTTAGCGATTTTAAAAGTTTTTTT
FYX044	GGAGCTAACCTGAGAAGTGTTTTTGTCCATCACGCA
FYX045	CTGGTTTCGAGCGCTCGAATCTCTAGAGGATC
FYX046	GCCGCCAGATTTACCGTTCCAGTAACATGGCT

FYX047	TAAAAATCAATAGAAAATTCAAACGCAATCCATAT
FYX048	AAACCACCAAATCCTTTGCCCGAAAATAGATA
FYX049	GTTGGCAATATTAACACCGCCTGCAGCAGAAG
FYX050	CTCCACATGGTGCCTAGGAA
FYX051	AACCCTCATTTGCGGGAGAAGCCTCAATAAAGTAGC
FYX052	AAAAATAATAATTCTGTATTACCGCCTTGCTGGTA
FYX053	CTCGTATTAGAAGGAGCGGAATTATATCAGAT
FYX054	TTTAAAGACGCTTAGGTCTGAGAGACTACTGATGCACTTTTTCA
FYX055	TCCAAGCAAGCCTCAGAGCCGCCACCAGCCAGAGCC
FYX056	TTTAATTGGTGAGAATAGAAAGGATGGGATTTTGCT
FYX057	AAAGGGAAGGAAGGGAAGAAAGCGAAAAGTGAGA
FYX058	TGGAACAATTGATGGTGGCGCGAGAAATTTAACCTT
FYX059	TCATGGAACTTGCCTGAGTAGAAGTAGCAATACTTCTTTG
FYX060	AGCGAAGGTAAATATTGACGCAGTAGCAAGGACAG
FYX061	GTATTCTAAGAACGCGATATCAACATT
FYX062	TTACTGTACTGGAAAGCGCAGTCTCTGACATT
FYX063	ACCTCCGGGAAAACATAGCGATAGTCGCTAT
FYX064	GGTCAGTTCAACATTAAGGAA
FYX065	CAAAAAGAAACAGGTTGATACGCCACCCTCAGAACCTGGCATGATAATA
FYX066	CAGTTTGAGGGGGGCACCGCTGGATAGCGAAA
FYX067	CAATTATTAAAGAACGTGG
FYX068	TAAATATTTCATCTGCAC
FYX069	GAAGGCTTAAGCAAATGCAC
FYX070	GTAAGAATGCTCAATCGTCTGAAAAAAAACGC
FYX071	CCCACGCATAACTATCGGTTTATCAGCTAAGGAGCC
FYX072	TAACGCCAGCATGCCTGAATGCAGAGAAGCAAA
FYX073	TAATTAATTAATGGAAACAGTACATCATTTAACAA
FYX074	CCTTTTACATCGGGAGATTCATTTCTTACAGAGATAA
FYX075	CTATGGTTGTTCTTTTCGAAAAAGCGACCGTGTGA
FYX076	TAATTAACTGAGTATAAACGAGGCTGAGACTCCTCGTCGA
FYX077	ATAAAGAACGGATTCGCCTGATTG
FYX078	AATCATAAATGCATCTGC
FYX079	ACAATTCCACACAACAAGCTGTTTCCAGACCGAAGA
FYX080	CAATATCAGAGAACAAGAATTGAGTTAACCTT
FYX081	TCGGTCGGGTAGCAACGGCTACAGAGGGCCAGGT
FYX082	TTAAAGTTAATGTACAGGAGCAACGCTACTAC
FYX083	GCGAAACAGGAACGAGGCGC
FYX084	TTCGTCTTTTCATAATCAAACACCACCCGTTTTTA
FYX085	GAACCAGAGCCACAGGCGGATAAGTGCCAAGA
FYX086	AATGAAACGGCCGGAAACCGATTGAGGGAGGGCCAAAGAC
FYX087	CCCAGACGAGCGTTGGCCGGTTGATAATCAGAAA
FYX088	GATTCAAAAATCTTTAGCGTCCACC
FYX089	AGCCCGGAAAACATGAAGTAA
FYX090	TTAGCCTTTAATGGGAATTAGCCCGAAATTGC
FYX091	TAATAAGCCAACGCTCAACAAATTACTAACCGGAGC

FYX092	ATAAAATGAAACAACGGG
FYX093	CACTAATGTATCATCGCCTGATAAATTTATCAA
FYX094	AATTAATGAGTAACATTATCATTT
FYX095	ACCGGAACCGCCTCCCTATAT
FYX096	AAGGCCGGATTTTTAGTGAA
FYX097	CGGAACGAGCTGAGGCTTGCAGGGCAACAACCATCG
FYX098	ATAGGTGGGAACTGGCCTTCCTGTAGCCCAGCTCATTAGTAAGATCATC
FYX099	GGGCTTGAATTCAAAAATCAATATGATATTCAAATTAATGGAGAGTCT
FYX100	ATAAAACAATTTTTGAATGGCTATCTGAAAGC
FYX101	TCAATAACAGTAGATTTACAGACCCGGAATAAGTTGTGTC
FYX102	AAATCTCCCGAATAATAATT
FYX103	CAGCCCTCATAGTTAGTTTCTGTAACAACTAA
FYX104	AGGACAAAATCAGAGAAGA
FYX105	TTGAGCATTAGAACATCAAGGAGCAAAAGAA
FYX106	AAATCCTCTGGCCTTGAAGGAATTAAAACCAAAAT
FYX107	AATCCAATGAAAATGTCAGAT
FYX108	TGGCGAGAAGCCCCCGATTTAGAGGCCCACTA
FYX109	CGTGAACCTCAAAGGGTAACTATATGTAAATGCCTTTTTA
FYX110	ACAACGGAGATTAACACTCATCTTTGACCACCAACCTAAA
FYX111	TGTGGCCAGTGCCAAGCTTGGGTTTTCCAAAA
FYX112	CATTTTGCTCCTTCGAGCTTCAAAGCGAACCTG
FYX113	GTACCGATAAGGGAACCGAACTGACCAACTTCCATGTTACTTAGCCAAGT
FYX114	TCATATTAAAAAAACTGATAGCCCTAAAATTC
FYX115	CAAATATTCACCGATCAGAGCCGCCACCTGCG
FYX116	AGCTCGCATTATTATCCTGGCAACACTGGAGG
FYX117	AAAAGGGCAATACATACATAAAGGCGCAGTATCCGC
FYX118	ATACATTTTCTTTAGGAGCACTAACAAA
FYX119	GTTTCTTTGACGCCAGTATATTAGGCAGCCAT
FYX120	CTGTCAACAGCTGAAATACCCTGTTTAGTACC
FYX121	AAACAGGGAAGCGCGCTAATATATGTGAGTGAACAAGG
FYX122	TATCGAGCCAGCAGCAAATGACATCGCCCGAGAACAAGAA
FYX123	AAAAAGAAAGATATACCAGTCAGTGCCCACAAAATT
FYX124	TTGCTCAGAGCATCACCGGGAACTGTCTTTCCTTACGCC
FYX125	GTTGCGCCTTCTTAAACAGC
FYX126	CGTGCCAGCTGCATTAAGGCGGTTTATATAATGAGTT
FYX127	TCTAAAGTTCGTCACCAGTACAAAAGGAACCCAT
FYX128	AGTCAGAATGGTAATAAGTTTTAACGG
FYX129	GAAGGGTTTTCATCAATATAATCCGAACAAAG
FYX130	AAATGTTAGAGCCGTCCGTTATTAAACCTCCCGCTATTTTGCA
FYX131	GCAACAGGTGGATTATTTACATTGATAGAACC
FYX132	TTTGATGACCCCCTGCCTATTTCG
FYX133	GATGGCAAAGAACCTACCATATCAAAACAGAA
FYX134	AGAACGTCAAAAAATAAGAAAATCATA
FYX135	AATAAAACGCAATTAAGACTTGGTGATT
FYX136	ACGCTATATTTAATTCTACGAGTGTTGTTCCAGTT

FYX137	AAGACCTGAAAAGGAATTTATAACCGAGGGCTA
FYX138	GTCAATAGTTTATTTCAATAAGAGCGTCAGAGGAGTA
FYX139	CTTCTGACTAGTCTTTAATGCGCGTACCGAACGAACCACCAACAGTGC
FYX140	AACTCATTGCCTCCGGAGAGGGTAGCTATTTTTGAGAGACAGTCTTGTT
FYX141	CTCCTAAAATGTTTACCAGACCGCC
FYX142	ACCCAGGCAAAGCATAAAGCTAAATCGGATGAC
FYX143	GCTTCTGTAAATCGCTTAGATTAGAAAAGTAGCCGAAC
FYX144	CACCACCGAAGCGCCCAATAACGTAACCTT
FYX145	GCATAAAGTGTACCGAGTAAGGTACCGACAGTAATACGACAATAATTA
FYX146	GTTTAATAGTAGTAGCATTTACAGGCAAGGCGCATACAA
FYX147	GGAGGTTTAGTAGTTAGCAAAAAGTTACCGAGAAAAAATC
FYX148	CCGCGCTTGCAAGTGTAGCGGTCAGGCGAACG
FYX149	AAGGTTATCTAAAATAGAGGATTTAGAAGTATTTCGACAA
FYX150	AAACAGCCATATAGCCTAATTTGCCAGTTAAATCAA
FYX151	TGTACCATTATACTTCTGGTT
FYX152	AACGGTACGCCAGAATACAGGAGGCCGATTAACACACCCG
FYX153	GGCCCTCGCTGGTTTGCGAACGCTGTTTAGGAAT
FYX154	ACTCCAACGATCACCCAAATCAAGTTCGGAACCCT
FYX155	TCATAGACACCAAAAAGAACGCCACCCTCGGATAT
FYX156	ACGAAACCAGAGCAGACTGT
FYX157	AGCTGGCGAGGGAAGGGACAGTTCAGTCCAATACCTC
FYX158	GGGACCATAAATACGCGCCTTAGCGACAAAAA
FYX159	GCAACATCGATAGCAGCACCTTCATCGGAATCAAT
FYX160	TGGACTGTAGCTTTTGTCACGGTGAATTATCACCGTCACCGGAATA
FYX161	GAACCAGAGGGAGTATCGGATAGGTCACGTTGGTGTAGATGGGGATT
FYX162	CACGCTGAAAACCCTCAATCAATATCTGGTCA
FYX163	TCTTCTCAGAACTAAGTAT
FYX164	TGGCCGAGCATGGAACAAG
FYX165	CGGGTATTAAACCTAATTTACAACA
FYX166	CTTTTGCTTTAACGATCGGTCTCCAGCC
FYX167	AGGAATTGAAAAAAAAGGCTCCAATGCTTTC
FYX168	GAGATGCCCAAATCAACGTGTAATACTTATATTTT
FYX169	AAATGCAAAGGTAAAGGATGGTTTTGCGATTTTGGGA
FYX170	TTGAGTAACAGGACGTTAAG
FYX171	GAAGCAGAACGAGTAGTCCC
FYX172	TATTCTGATAGGTGTATCAC
FYX173	AGGGACGCGCCATTTCAT
FYX174	GAGGTGAATGACAATGAAGTTAAAGGCCGCTTTACCCTC
FYX175	GTTTGCCAGTCATAGCCCCC
FYX176	AACAGTTTTGATTGCCCGGCGCCAGGGTG
FYX177	TACCAACAACATACAATAGAGTAGATTAAGTTGGG
FYX178	GATTAGTTGACTTGCGGGAGGTTT
FYX179	TCATTAAAACCTCAGAGAATTAGCAAAATTAAGGTGG
FYX180	ACCTTGCTGAACCTCAACTAATCCAATAGCATCCG
FYX181	ACGAAGAGAATAAACGCCAACGTTAGAAGCGCGTA

FYX182	AGCTTTCCACGACGACGGTA
FYXh01	CCTACGAGTCAGTCCTTTCAGAAGCAGACTTTTAAAACTTGAGC
FYXh02	AGAAAAATCTACGTTAATATTCATCTTCACACTACT
FYXh03	CCTACGAGTCAGTCCTTATCAAGAGTAATCTTGAGGC
FYXh04	CCTACGAGTCAGTCCTTAAACGAACTAACGGAATCAGCGGAAAACAAAT
FYXh05	CCTACGAGTCAGTCCTTTGCAAAAGAAGTTTTGTCGTC
FYXh06	CATGTTTTAAATATTCATCTTCACACTACT
FYXh07	CGGAGACTTCAAATATCGCGTTCATCTTCACACTACT
FYXh08	TACCACATTCAACTAATGCTTCATCTTCACACTACT
FYXh09	ATAAATATTCATTCATCTTCACACTACT
FYXh10	CCTACGAGTCAGTCCTTATTGTGAATTACCTTAAATT
FYXh11	AAACCTTTAATCTTCATCTTCACACTACT
FYXh12	TGGCGAACGGTGTAGTTTGACCATTAGATTCATCTTCACACTACT
FYXh13	AGCGAGAGGCTTTTTCATCTTCACACTACT
FYXh14	CCTACGAGTCAGTCCTTTACATTTCGCAAATGG
FYXh15	CCTACGAGTCAGTCCTTAGATACATAACGC
FYXh16	ACCCTGACTATTATAGTTCATCTTCACACTACT
FYXh17	CCTACGAGTCAGTCCTTTTGAATCCCCCTCAAA
FYXh18	CCTACGAGTCAGTCCTTAAGGCTTGCCCTGACGTGCTCATTCTCAGTACCCA
FYXh19	CCTACGAGTCAGTCCTTAGGTCATTTTTGCGGATGGC
FYXh20	CCCTCATTTTCAGGGACAGATTGACCTTCTTCATCTTCACACTACT
FYXh21	AACGCCTGTAGCATTCCTTGCAGTGAATTTCATCTTCACACTACT
FYXh22	CCTACGAGTCAGTCCTTTGCAACTAAAGTACGGTGTC
FYXh23	CCTACGAGTCAGTCCTTTTTTTAATTTTGATAAGTTCATCTTCACACTACT
FYXh09	ATAAATATTCATTCATCTTCACACTACT

Table S6. Names and sequences of DNA oligonucleotides for preparing NP-I^{DNA} and NP-O^{DNA}. The oligonucleotides replace the indicated staple strands from Table S4 to position the 3'-terminal 20 nt-long single stranded overhang (sequence in bold) within or outside the pore lumen.

Replacing	ID	Sequence 5' → 3'
FYX070	Mouth-1	GTAAGAATGCTCAATCGTCTGAAAAAAAACGCTTT **GGAGTATTGCGGAGGAAGGT**
FYX076	Inside-2	ACTGAGTATAAACGAGGCTGAGACTCCTCGTCGATTT **GGAGTATTGCGGAGGAAGGT**

Table S7. Sequences of AF647-modified DNA oligonucleotides used for the fluorescent labeling of DNA nanopores. The oligonucleotides replace the indicated staple strands (Table S4) and carry an additional 15 nt-long 3'-terminal overhang (sequence in bold) to enable hybridization to complementary oligonucleotide ^{AF647}oligo.

Replacing	Sequence 5' → 3'
FYX049	GTTGGCAATATTAACACCGCCTGCAGCAGAAGTTT**ATCAGAGATCAGCATACA**

FYX053	CTCGTATTAGAAGGAGCGGAATTATATCAGATTTT**ATCAGAGATCAGCATACA**
FYX059	TCATGGAACTTGCCTGAGTAGAAGTAGCAATACTTCTTTGTT**ATCAGAGATCAGCATACA**
FYX063	ACCTCCGGGAAAACATAGCGATAGTCGCTATTTT**ATCAGAGATCAGCATACA**
FYX077	ATAAAGAACGGATTCGCCTGATTGTTT**ATCAGAGATCAGCATACA**
FYX100	ATAAAACAATTTTTGAATGGCTATCTGAAAGCTTT**ATCAGAGATCAGCATACA**
FYX108	TGGCGAGAAGCCCCCGATTTAGAGGCCCACTATTT**ATCAGAGATCAGCATACA**
FYX148	CCGCGCTTGCAAGTGTAGCGGTCAGGCGAACGTTT**ATCAGAGATCAGCATACA**
FYX149	AAGGTTATCTAAAATAGAGGATTTAGAAGTATTTCGACAATT**ATCAGAGATCAGCATACA**
FYX162	CACGCTGAAAACCCTCAATCAATATCTGGTCATTT**ATCAGAGATCAGCATACA**
^{AF647}oligo	/5Alex647N/TGTATGCTGATCTCTGAT

Table S8. Sequence of Alexa Fluor 488 and Cy5 labeled oligonucleotides used to form a duplex to determine extent of red and green confocal volume overlap.

**ID**	**Sequence 5' → 3'**
^{Alexa488}oligo^{Cy5}	/5Cy5/CGTACGCGGAATACTTCGATT/3AlexaF488N/
oligo^{comp}	TCGAAGTATTCCGCGTACGTT

4. Supplementary Figures

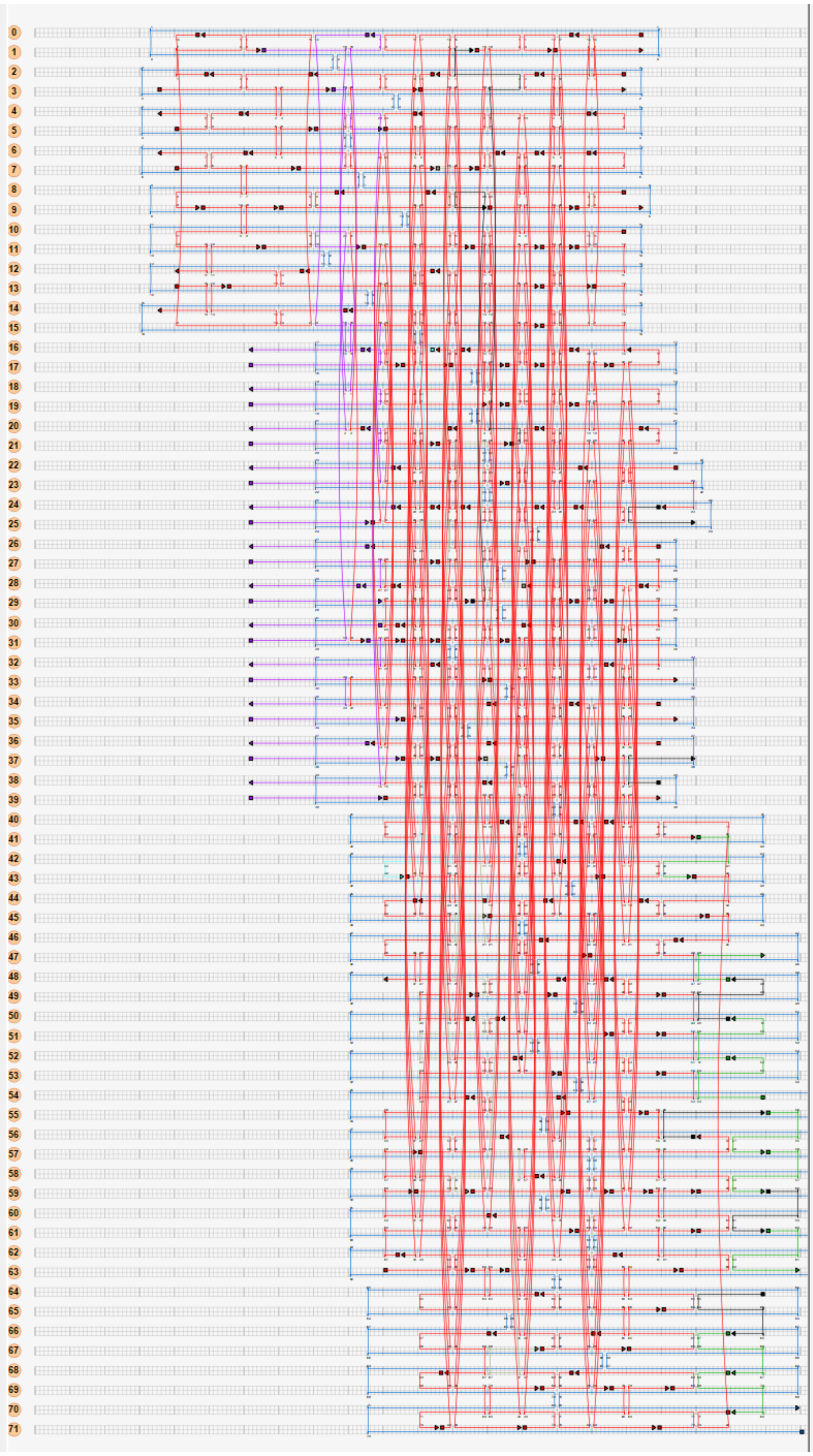

Figure S1. 2D connectivity map of NP designed with caDNAno. The scaffold strand is shown in blue and the staple strands are in red, purple, and green; the latter are used for attaching AF647-labled DNA oligonucleotides. 5' and 3' termini of DNA strands are represented by squares and triangles, respectively. The duplex numbering is shown on the left side and corresponds to the numbers in Figure S2.

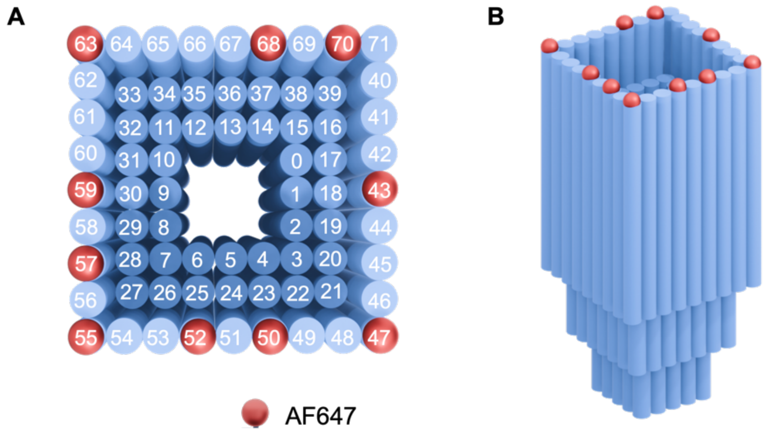

Figure S2. Location and numbering of fluorophores on a 2D duplex map and 3D model of NP. (A) The numbering of duplexes in NP corresponds to the duplexes in Figure S1. Red dots represent the location of Alexa Fluor 647 fluorophores. (B) 3D model of the NP decorated with 10 Alexa Fluor 647 dyes.

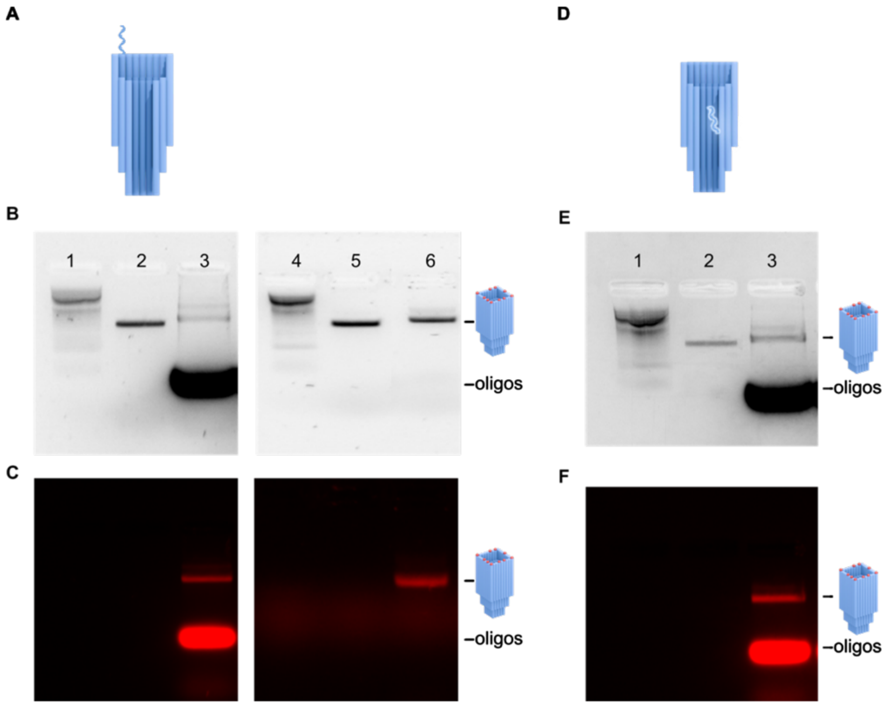

Figure S3. Agarose gel picture showing pore assembly as well as fluorescent labeling of the two pore constructs NP-O^{DNA} and NP-I^{DNA}. (A) Schematic illustration of NP-O^{DNA}. (B) 1 % Agarose gel electrophoretic analysis on the formation of NP-O^{DNA}. Lanes from left to right; (1) 1 kb ladder, (2) M13mp18 scaffold, (3) assembled non-purified NP-O^{DNA}, (4) 1 kb ladder, (5) M13mp18 scaffold, (6), assembled NP-O^{DNA} after gel purification and removal of excess staple strands. The DNA bands were stained with Gel Red. (C) Gel images visualized for AF647 to confirm fluorescent labeling of NP-O^{DNA}. (D) Schematic illustration of NP-I^{DNA}. (E) 1% Agarose gel electrophoresis analysis on the formation of NP-I^{DNA}. (D) Confirmation of the fluorescent labelling (red signal) of the NP-I^{DNA}. Lanes from left to right: (1) 1 kb ladder, (2) M13mp18 scaffold, (3) assembled NP-I^{DNA}.

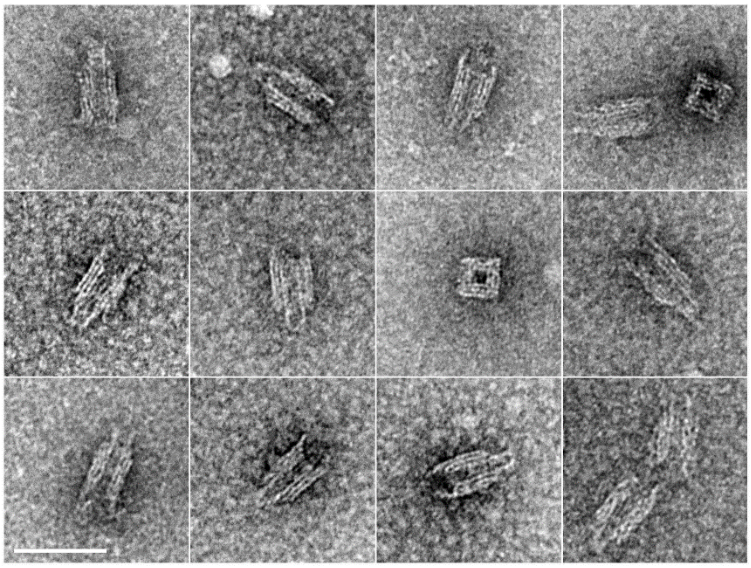

Figure S4. TEM images of negatively stained nanopore NP-N without DNA receptor sites. Scale bar, 100 nm.

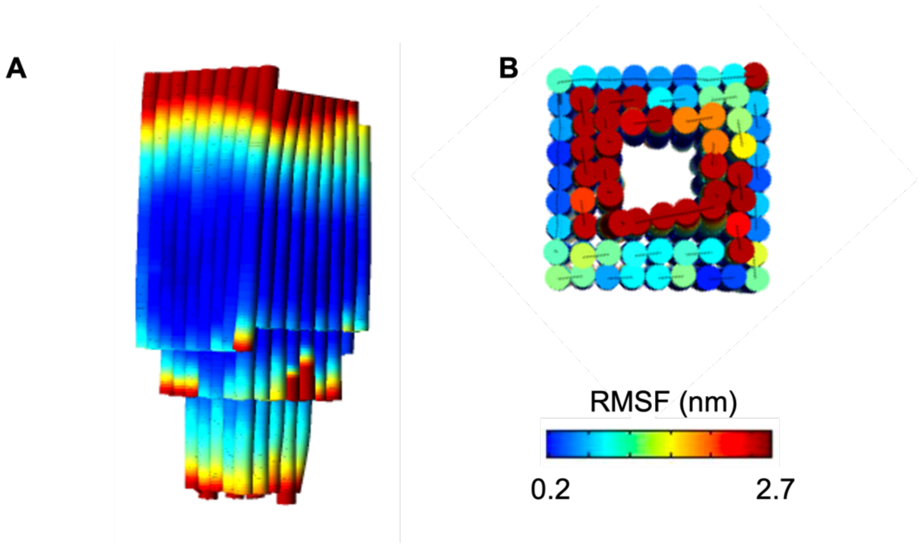

Figure S5. Thermal fluctuation analysis of NP. CanDo analysis[7] of NP in (A) front view and (B) top view, indicating high stability of the core (blue) with a root mean square fluctuation (RMSF) of 0.2 nm and higher flexibility towards the ends of the structure (red) of up to 2.7 nm due to presence of single-stranded loops.

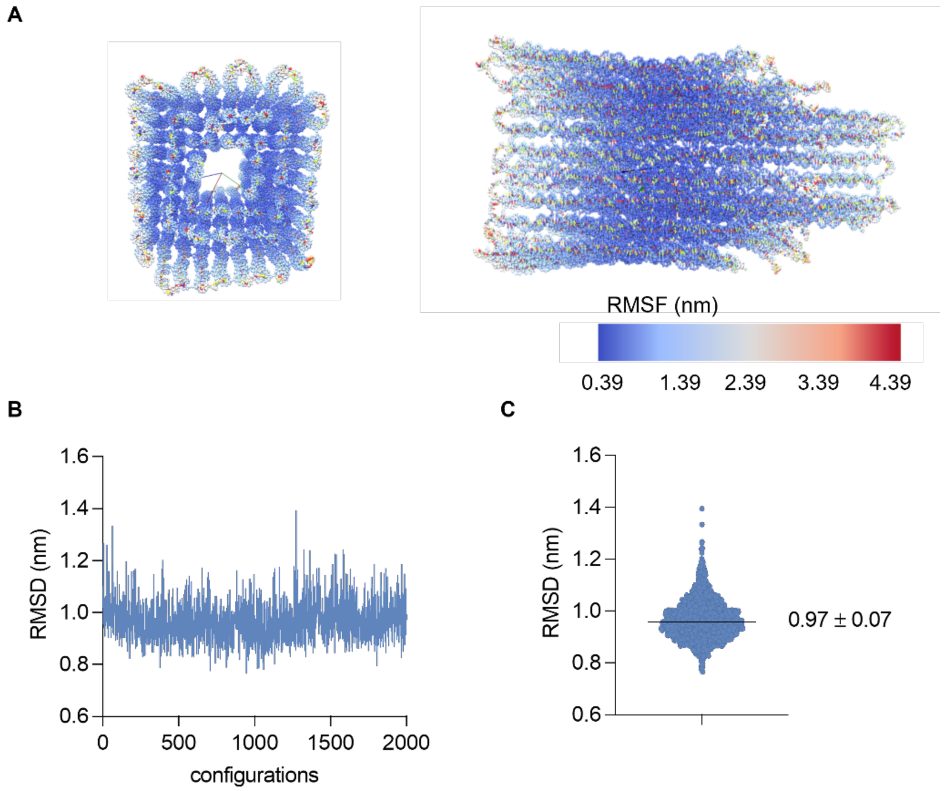

Figure S6. oxDNA simulations of DNA nanopore. (A) Color-coded root mean square fluctuation (RMSF) of the NP in solution, obtained by oxDNA simulation. RMSF is obtained by calculating the mean structure and computing per-nucleotide root mean square fluctuation from the mean. (B) Root mean square deviation (RMSD) of the NP for 2000-time steps. (C) Distribution of all RMSD for the different timesteps and mean value of 0.97 ± 0.07 nm. The coarse-grained modelling of the NP was performed using the oxDNA force field[8]. Starting conditions were obtained by transforming the caDNAno[9] file to oxDNA format via the TacoxDNA[10] converter. After relaxation, equilibrium simulations were carried out for 10^9 oxDNA simulation steps at 20°C and a salt concentration of one. RMSF and mean were calculated via the oxDNA server.

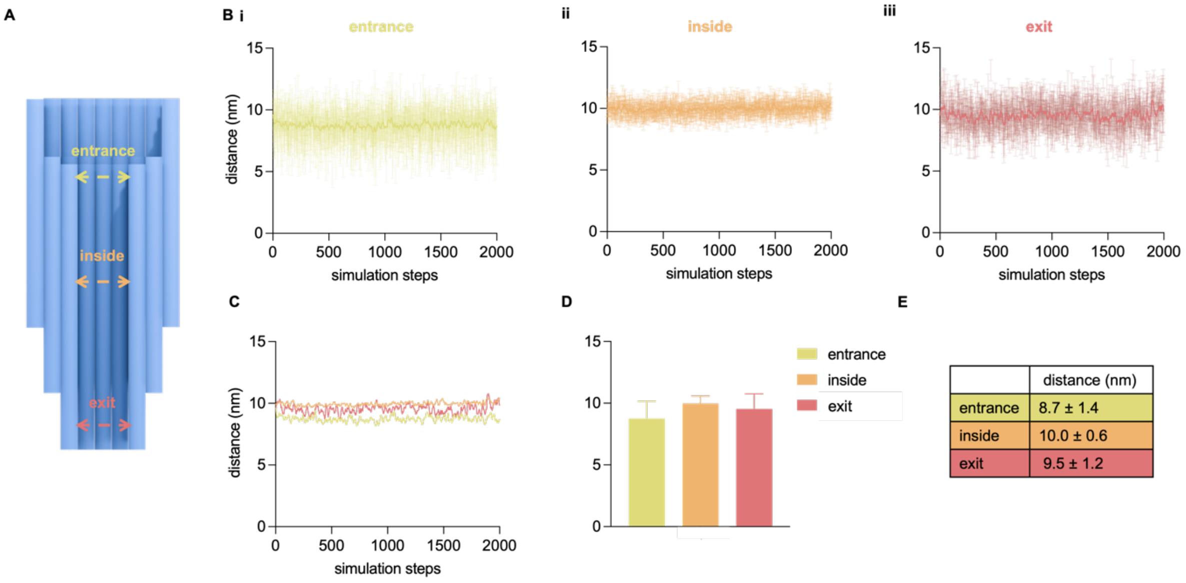

Figure S7. Analysis of oxDNA simulations. (A) Render of the NP lumen highlighting 3 sections (entrance, inside and end) which were used to calculate the lumen diameter over time. (B) Distance between opposing lumen enclosing duplexes at the (i), entrance (ii) inside and (iii) end. Averages of 4 simulations per graph, smoothed average shown. (C) Average smoothed trace of lumen diameter over time (2000 timesteps). (D) Average diameter for entrance, inside and end ± stdev. (E) Numerical value for the diameter in nm, mean ± standard deviation, n= 8000 datapoints.

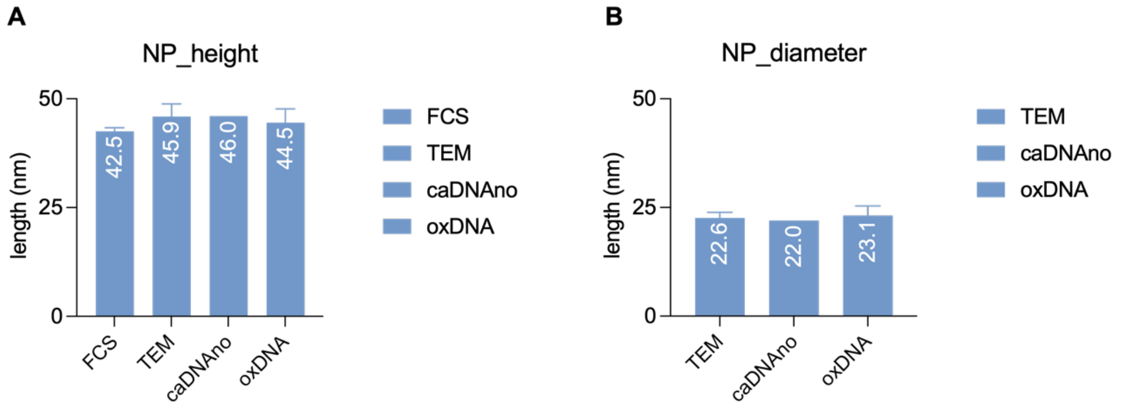

Figure S8. Comparison of NP dimension obtained via FCS, TEM, caDNAno and oxDNA.

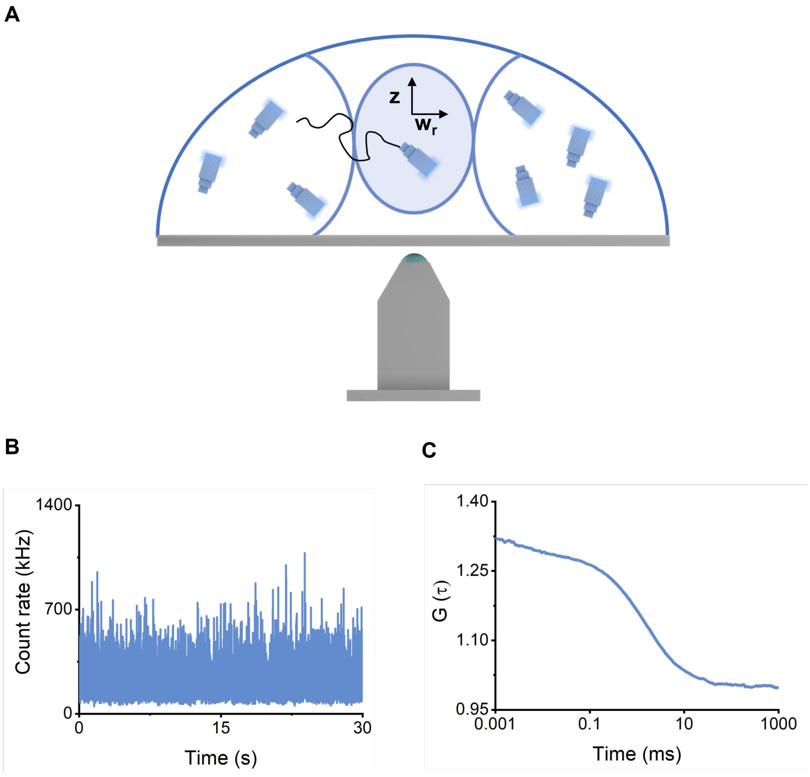

Figure S9. Fluorescence correlation spectroscopic analysis of purified NP yielding molecular brightness, diffusion time, and concentration of the NP. (A) Schematic illustration of fluorescent DNA nanopores diffusing through the red detection volume of a confocal microscope giving rise to intensity fluctuations shown in (B). (C) Correlation curve calculated as the autocorrelation of the intensity fluctuations (B) measuring the self-similarity of the fluorescence signal. Fit of correlation curve yields the diffusion time τ_{D}. Based on the known lateral radius of the red confocal volume, ω_{r}, which was determined prior to the measurement using free dye Alexa Fluor 647 maleimide, and the measured diffusion time (τ_{D}), the diffusion coefficient (D) of the DNA NP was calculated to be 5.02 ± 0.44 $\mu m^2 s^{-1}$. Subsequently, the hydrodynamic diameter (dH) of the DNA pore was evaluated using the Stokes-Einstein equation (Eq. S4), yielding a value of 48.0 ± 4.6 nm.

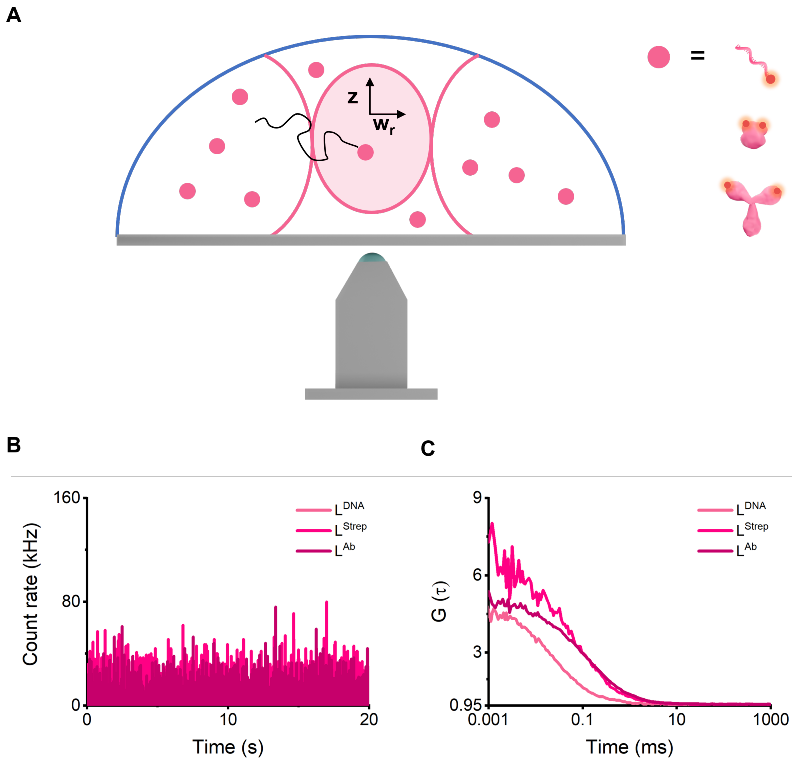

Figure S10. Fluorescence correlation spectroscopic analysis of Alexa Fluor 488-labeled ligands L^{DNA}, L^{Strep}, and biotin L^{Ab}, yielding respective molecular brightness and diffusion times. (A) Schematic illustration of fluorescent ligands, diffusing through the green detection volume of a confocal microscope giving rise to intensity fluctuations shown in (B). (C) Correlation curve calculated as the autocorrelation of the intensity fluctuations (B). Fits to correlation curves yield diffusion coefficients of 163 ± 51 $\mu m^2 s^{-1}$ for L^{DNA}, 91.3 ± 11.2 $\mu m^2 s^{-1}$ for L^{Strep}, and 41.2 ± 1.6 $\mu m^2 s^{-1}$ for L^{Ab}.

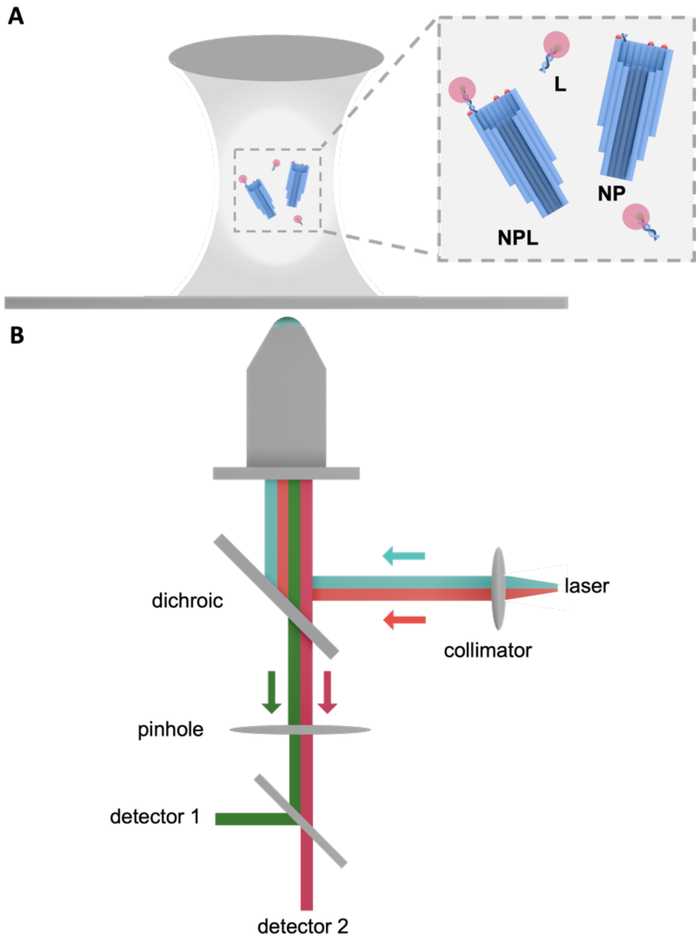

Figure S11. Illumination path for the detection of nanopore NP, ligands L, and NP•L complexes. (A) Schematic drawing of fluorescent species NP, L and NP•L diffusing through the femtoliter-sized confocal volume. (B) NP, L and NP•L are illuminated with two spectrally distinct laser lines (488 nm and 630 nm). Excitation light is separated from emission light by a dichroic mirror. Emission light then passes the pinhole and is then further separated by a second dichroic mirror to reach their respective detectors.

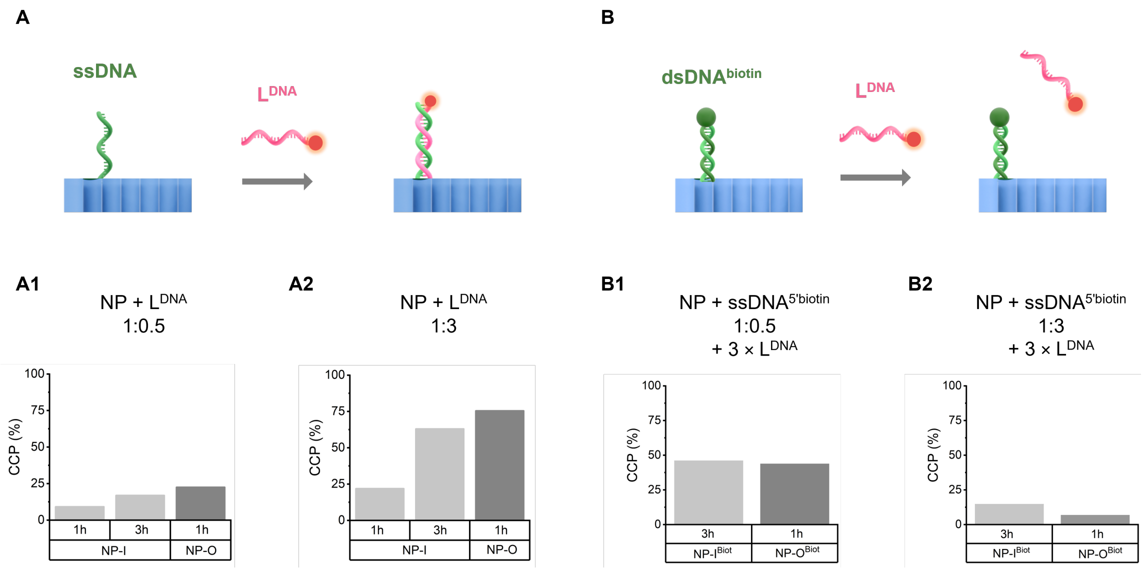

Figure S12. Binding of biotin-tagged oligonucleotide to receptor DNA within NP-O^{DNA} and NP-I^{DNA}. (A) Schematic drawing of the interaction between fluorophore-tagged L^{DNA} and a DNA-modified nanopore and (B) between fluorophore-tagged L^{DNA} and a biotin-tagged nanopore. (A1-B2) Histograms summarizing the extent of L^{DNA} binding to (A1, A2) NP-O^{DNA} and NP-I^{DNA} and (B1, B2) NP-O^{Biot} and NP-I^{Biot} preparations containing varying amounts of ssDNA$^{5'biotin}$ and L^{DNA}. The molar ratios of the ligands and nanopores as well as the incubation times are described in the figure panels.

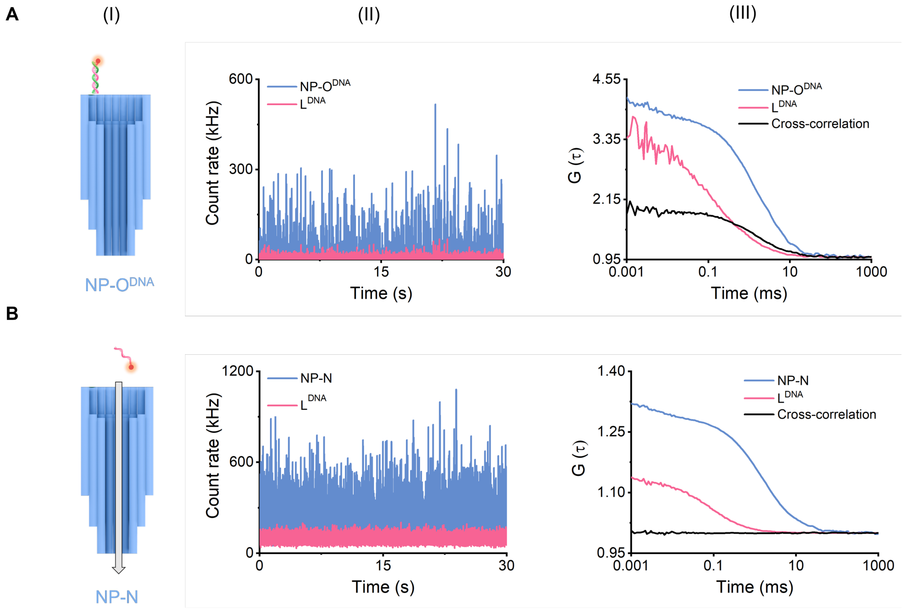

Figure S13. Control for excluding non-specific binding of L^{DNA} to NP. (A-I, B-I) Schematic illustrations of NP-O^{DNA} and NP-N without any receptor. (A-II) Fluorescence intensity fluctuations in the red and green channel of a reaction mixture containing NP-O^{DNA} and L^{DNA}. (B-II) Fluorescence intensity fluctuations in a reaction mixture of NP-N and L^{DNA}. (A-III) Upon NP-O^{DNA} and L^{DNA} binding and co-diffusion, cross-correlation can be detected. (B-III) No cross-correlation can be observed for NP-N and L^{DNA}.

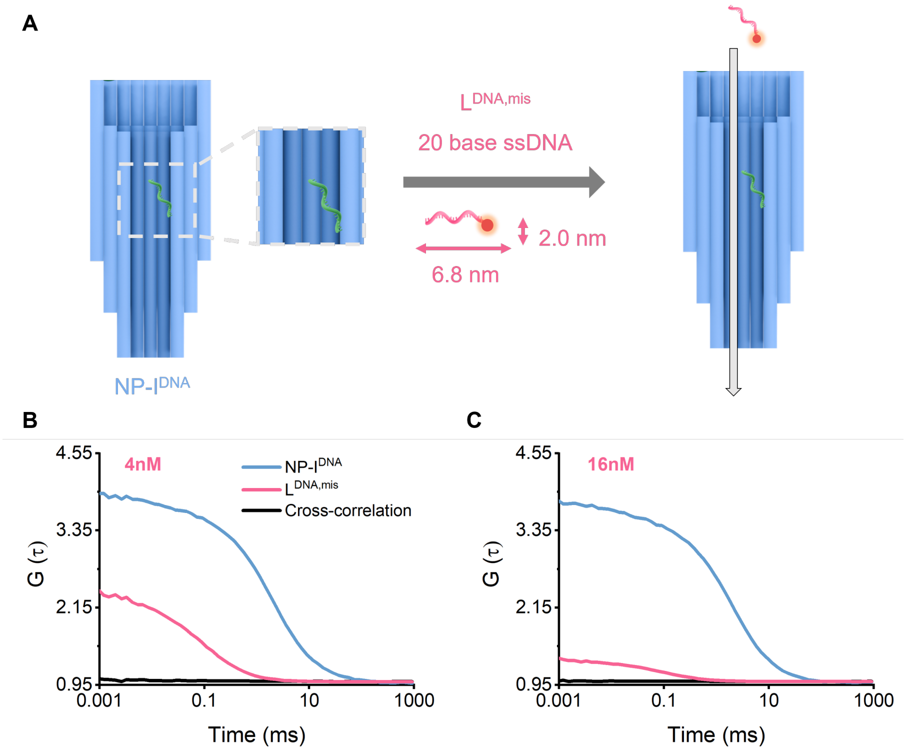

Figure S14. Control experiment showing that no confinement-induced co-diffusion occurs using L^{DNA} containing 11 mismatches. (A) Schematic representation of the receptor site composed of a 20 nucleotide long single-stranded DNA (ssDNA) overhang. L^{DNA} was added, but this time containing 11 mismatches, resulting in a sequence that is no longer fully complementary. Under these conditions, the ligand is not expected to bind to the receptor and should diffuse freely through the pore lumen, without being trapped in the confined pore lumen. No cross-correlation was detected at ligand concentrations of either (B) 4 nM or (C) 16 nM. The concentration of NP-I^{DNA} was 1.5 nM in all reaction mixtures (B+C). The lack of any cross-correlation signal confirms the absence of binding to the receptor site and ruling out false-positive cross-correlation due to co-diffusion of the ligand in the confined pore lumen.

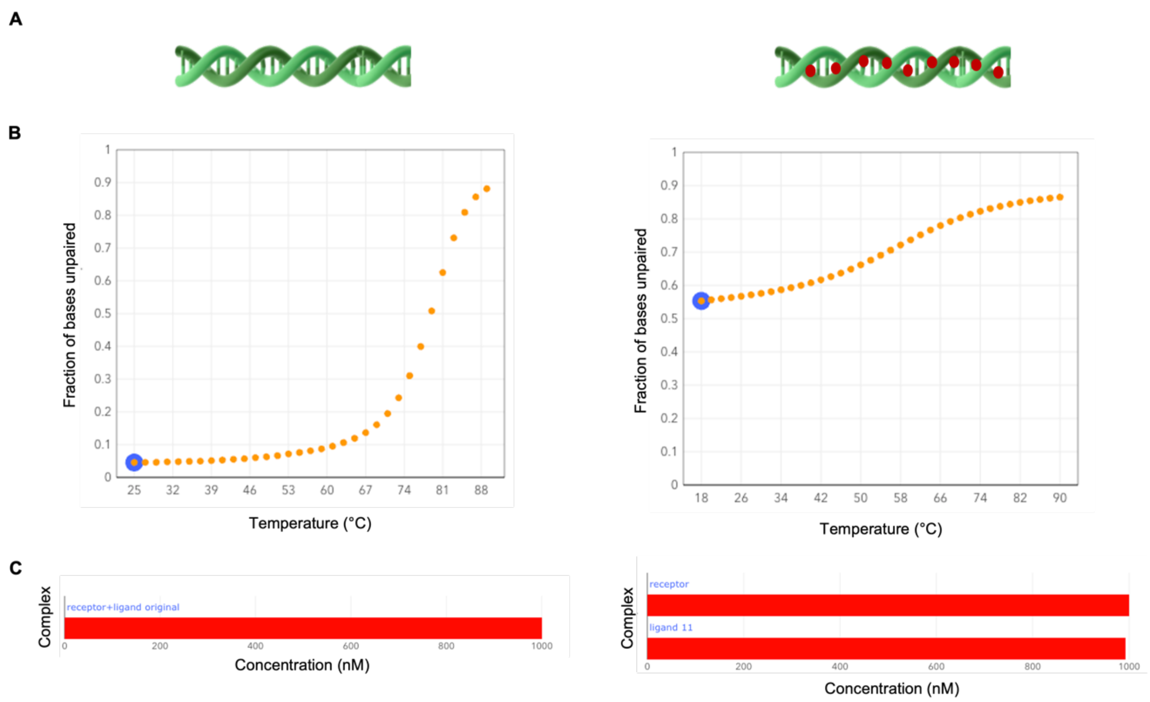

Figure S15. NUPACK analysis[11] of DNA duplex formation for fully complementary L^{DNA} and L^{DNA} containing 11 mismatches. (A) Schematic illustration of receptor hybridization with a fully complementary ligand (left) and with a ligand containing 11 mismatches (right). (B) Thermodynamic simulations of base-pairing across temperature. The fully complementary duplex (left) remains largely paired at room temperature (~6% unpaired at 25 °C) and shows a sharp melting transition, indicating stable hybridization. The mismatched duplex (right) is already ~56% unpaired at 25 °C and denatures gradually, indicating instability. (C) Predicted complex formation at 25 °C as a function of concentration. The complementary strands (left) show duplex formation at across the entire tested ligand concentration range (up to 1000 nM), while the mismatched strands (right) show no significant complex formation, confirming lack of binding.

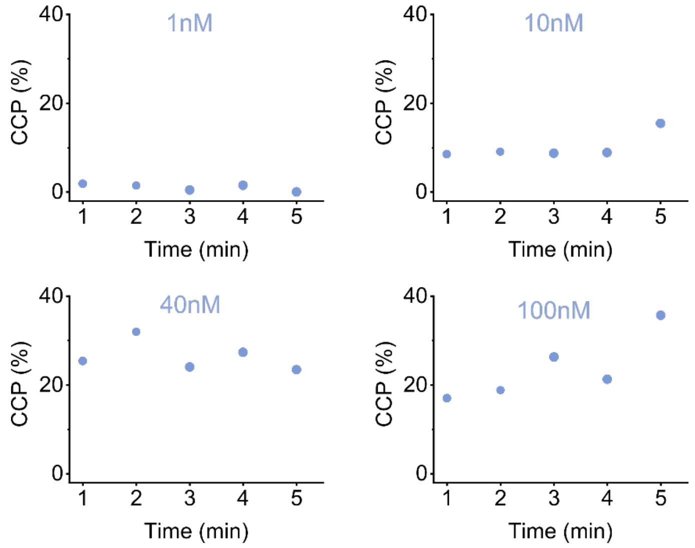

Figure S16. Fast equilibration of $NP\text{-}I^{Biot}$ binding to L^{Ab}. The formation of the binary nanopore-ligand complexes reaches plateau after 1 min of incubation of 2 nM $NP\text{-}I^{Biot}$ with L^{Ab} at concentrations equal to 1 nM, 10 nM, 40 nM and 100 nM.

References

(1) Erokhova, L.; Horner, A.; Ollinger, N.; Siligan, C.; Pohl, P. The Sodium Glucose Cotransporter SGLT1 Is an Extremely Efficient Facilitator of Passive Water Transport. *J. Biol. Chem.* **2016**, 291, 9712–9720.

(2) Elson, L. E.; Douglas, M. Fluorescence Correlation Spectroscopy. I. Conceptual Basis and Theory. *Biopolymers* **1974**, 13, 1–27.

(3) Bacia, K.; Haustein, E.; Schwille, P. Fluorescence Correlation Spectroscopy: Principles and Applications. *Cold Spring Harb Protoc.* **2014**, 2014, 709–725.

(4) Werner, S.; Ebenhan, J.; Haupt, C.; Bacia, K. A Quantitative and Reliable Calibration Standard for Dual-Color Fluorescence Cross-Correlation Spectroscopy. *Chemphyschem.* **2018**, 19, 3436–3444.

(5) Krüger, D.; Ebenhan, J.; Werner, S.; Bacia, K. Measuring Protein Binding to Lipid Vesicles by Fluorescence Cross-Correlation Spectroscopy. *Biophys. J.* **2017**, 113, 1311–1320.

(6) Jarmoskaite, I.; AlSadhan, I.; Vaidyanathan, P. P.; Herschlag, D. How to Measure and Evaluate Binding Affinities. *Elife.* **2020**, 9, 1–34.

(7) Kim D.; Kilchherr F.; Dietz H.; Bathe M.; Quantitative prediction of 3D solution shape and flexibility of nucleic acid nanostructures, *Nucleic Acids Res.*, **2012**, *40*, 2862–2868.

(8) Poppleton E.; Romero R.; Mallya A.; Rovigatti L.; Šulc P. OxDNA.org: a public webserver for coarse-grained simulations of DNA and RNA nanostructures. *Nucleic Acids Res.* **2021**, *49*, 491-498.

(9) Douglas SM.; Marblestone AH.; Teerapittayanon S.; Vazquez A.; Church GM.; Shih WM. Rapid prototyping of 3D DNA-origami shapes with caDNAno. *Nucleic Acids Res.* **2009**, 37, 5001-6.

(10) Suma A.; Poppleton E.; Matthies M.; Šulc P.; Romano F.; Louis AA.; Doye JPK.; Micheletti C.; Rovigatti L. TacoxDNA: A user-friendly web server for simulations of complex DNA structures, from single strands to origami. *J Comput Chem.* **2019**, *40*, 2586-2595.

(11) Zadeh JN.; Steenberg CD.; Bois JS.; Wolfe BR.; Pierce MB.; Khan AR.; Dirks RM.; Pierce NA. NUPACK: Analysis and design of nucleic acid systems. *J Comput Chem*. **2011**, *32*, 170-3.
